# Case-area targeted interventions during a large-scale cholera epidemic: A prospective cohort study in Northeast Nigeria

**DOI:** 10.1371/journal.pmed.1004404

**Published:** 2024-05-10

**Authors:** Jennifer OKeeffe, Lindsay Salem-Bango, Michael R. Desjardins, Daniele Lantagne, Chiara Altare, Gurpreet Kaur, Thomas Heath, Kanaganathan Rangaiya, Patricia Oke-Oghene Obroh, Ahmadu Audu, Baptiste Lecuyot, Timothée Zoungrana, Emmanuel Emeka Ihemezue, Solomon Aye, Mustafa Sikder, Shannon Doocy, Qiulin Wang, Melody Xiao, Paul B. Spiegel

**Affiliations:** 1 Center for Humanitarian Health, Department of International Health, Johns Hopkins Bloomberg School of Public Health, Baltimore, Maryland, United States of America; 2 Spatial Science for Public Health Center, Department of Epidemiology, Johns Hopkins Bloomberg School of Public Health, Baltimore, Maryland, United States of America; 3 Lancon Environmental, LLC, Somerville, Massachusetts, United States of America; 4 Action contre la Faim, Paris, France; 5 Action contre la Faim, Abuja, Nigeria; 6 Solidarités International, Paris, France; 7 Solidarités International, Maiduguri, Nigeria

## Abstract

**Background:**

Cholera outbreaks are on the rise globally, with conflict-affected settings particularly at risk. Case-area targeted interventions (CATIs), a strategy whereby teams provide a package of interventions to case and neighboring households within a predefined “ring,” are increasingly employed in cholera responses. However, evidence on their ability to attenuate incidence is limited.

**Methods and findings:**

We conducted a prospective observational cohort study in 3 conflict-affected states in Nigeria in 2021. Enumerators within rapid response teams observed CATI implementation during a cholera outbreak and collected data on household demographics; existing water, sanitation, and hygiene (WASH) infrastructure; and CATI interventions. Descriptive statistics showed that CATIs were delivered to 46,864 case and neighbor households, with 80.0% of cases and 33.5% of neighbors receiving all intended supplies and activities, in a context with operational challenges of population density, supply stock outs, and security constraints.

We then applied prospective Poisson space-time scan statistics (STSS) across 3 models for each state: (1) an unadjusted model with case and population data; (2) an environmentally adjusted model adjusting for distance to cholera treatment centers and existing WASH infrastructure (improved water source, improved latrine, and handwashing station); and (3) a fully adjusted model adjusting for environmental and CATI variables (supply of Aquatabs and soap, hygiene promotion, bedding and latrine disinfection activities, ring coverage, and response timeliness). We ran the STSS each day of our study period to evaluate the space-time dynamics of the cholera outbreaks.

Compared to the unadjusted model, significant cholera clustering was attenuated in the environmentally adjusted model (from 572 to 18 clusters) but there was still risk of cholera transmission. Two states still yielded significant clusters (range 8–10 total clusters, relative risk of 2.2–5.5, 16.6–19.9 day duration, including 11.1–56.8 cholera cases). Cholera clustering was completely attenuated in the fully adjusted model, with no significant anomalous clusters across time and space. Associated measures including quantity, relative risk, significance, likelihood of recurrence, size, and duration of clusters reinforced the results. Key limitations include selection bias, remote data monitoring, and the lack of a control group.

**Conclusions:**

CATIs were associated with significant reductions in cholera clustering in Northeast Nigeria despite operational challenges. Our results provide a strong justification for rapid implementation and scale-up CATIs in cholera-response, particularly in conflict settings where WASH access is often limited.

## Abstract 2

Jennifer OKeeffe and colleagues prospectively study the impact of case-area targeted interventions (CATIs) in Northeast Nigeria during the 2021 cholera outbreak to better understand how to successfully deploy and scale-up CATIs as a core response to future outbreaks.

## Author summary

**Why was this study done?:**

- Case-area targeted interventions (CATIs)—whereby teams deliver a mixture of health and water, sanitation, and hygiene (WASH) interventions to cholera case households and neighboring households within a predetermined radius—have become an increasingly common strategy to respond to cholera outbreaks.
- Existing research has suggested that CATIs can reduce the number of cholera cases, among other positive effects. However, such evidence stems from (1) modeling studies; (2) retrospective studies; or (3) controlled trials.
- A gap exists in prospectively studying CATIs in uncontrolled environments, a limitation that has hindered the deployment and scaling-up of CATIs as a core response to cholera outbreaks.

**What did the researchers do and find?:**

- This study followed CATI teams during a large-scale cholera outbreak in Northeast Nigeria in 2021, collecting data on interventions provided, geospatial coordinates, demographics, and existing WASH infrastructure.
- Researchers used novel spatiotemporal methods to prospectively analyze the clustering of cholera cases in the presence of CATIs.
- Findings highlight that CATIs were associated with a reduction in cholera clusters during the outbreak; clusters were fewer and smaller, occurred for shorter duration, and were less likely to reoccur in the presence of CATIs.

**What do these findings mean?:**

- CATIs can reduce cholera transmission in fragile settings, despite challenges such as high population density, supply stock outs, and security constraints.
- The study’s novel methods, comprehensive scope, and expansion of previous research collectively strengthen our understanding of the value and applicability of CATIs in conflict-affected settings and supports their continued use and scale-up.
- Key limitations of this study include selection bias, remote data monitoring, and the lack of a control group.

## Introduction

Cholera has been described as “a disease of inequity that strikes the world’s poorest and most vulnerable people [1].” After decades of progress, the world is facing an upsurge in cholera, including larger and more outbreaks, high mortality rates, and spread to previously cholera-free areas [2]. From 2021 to 2022, recorded cholera cases more than doubled from 223,370 to 472,697 [3]. Additionally, the number of countries experiencing cholera in 2022 rose with 13 previously cholera-free countries reporting new outbreaks [2,4]. Global case fatality rates (CFR) were the highest in over a decade, at 1.9% on average (and up to 2.9% in Africa), surpassing the maximum recommended threshold of <1% [2]. Climate change and political instability have been identified as main drivers of this upsurge [2].

Fragile settings have been linked to higher risk of cholera epidemics due to inadequate water and sanitation, high population density, weakened health services, inadequate surveillance systems, and poor health access [5,6]. The largest epidemics in recent history have occurred in humanitarian settings: Haiti in 2010 with >600,000 cases [7] and Yemen in 2017 with >1 million cases [8]. With the climate emergency and humanitarian crises threatening cholera mitigation efforts worldwide, there is a need for improved evidence-based interventions, particularly in conflict-affected settings that pose specific challenges for response efforts.

A growing body of research has highlighted that proximity to a cholera case is a risk factor for infection [9–11]. Household contacts have approximately double the infection risk compared to those exposed from community water sources [12], and individuals living within 50 m of a cholera case had 23 to 56 times the risk of infection than those living further away [11]. Subsequent studies have suggested that more targeted interventions may be effective in cholera outbreak response [13–17]. Thus, the Global Task Force on Cholera Control now recommends targeted interventions in hotpots over blanket programs, and case-area targeted interventions (CATIs) have become increasingly common [18]. During CATIs, teams deliver a mixture of health and water, sanitation, and hygiene (WASH) interventions to cholera case households and neighboring households within a predetermined radius. Scoping reviews on CATIs suggest they may be effective in reducing cholera transmission by reducing case-cluster sizes, decreasing infection among household contacts, and reducing overall transmission [19–21]. However, previous research on reducing cholera transmission with CATIs has been retrospective [22,23], based on modeling [24], or occurred in controlled settings which may not translate to real-world effectiveness [25]. A gap exists in prospectively analyzing CATI’s ability to attenuate incidence, particularly in conflict-affected settings.

We conducted a prospective observational cohort study in the conflict-affected states of Borno, Adamawa, and Yobe, Nigeria from September through December 2021. That year, Nigeria experienced Africa’s largest cholera epidemic in 20 years, accounting for 50% of cases (*n* = 111,062) and 87% (*n* = 3,604, CFR = 3.2%) of deaths globally [26]. The epidemic was concentrated in Northeast Nigeria, which has experienced prolonged insecurity that intensified the population’s vulnerability to cholera and hindered response efforts. This study aimed to investigate CATI association on cholera transmission in a real-world, uncontrolled environment using a novel spatial-temporal cluster detection approach.

## Methods

### Study design and setting

**Fig 1:**
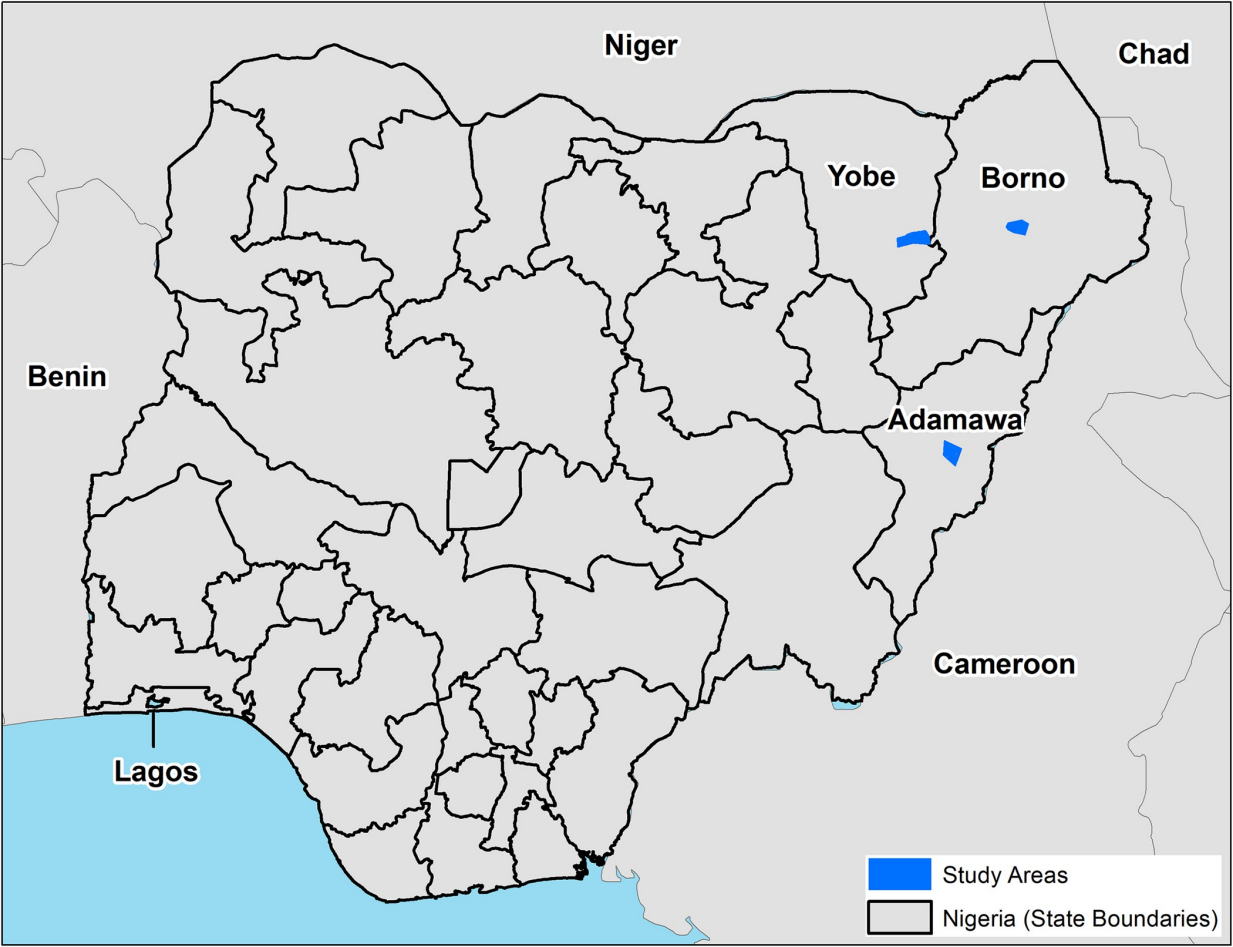
CATI response area in Borno, Adamawa, and Yobe states, Nigeria, Sept 15–Dec 25, 2021. The general study areas, covered with 150m^2^ grid cells, are shown in dark blue. Maps were created in ArcPro Version 3.1.2 [27]. The basemap is from the “World Imagery” layer provided by ESRI and can be found here: https://www.arcgis.com/home/item.html?id=10df2279f9684e4a9f6a7f08febac2a9 [28]. ArcPro is the intellectual property of Esri and is used herein under license. Copyright Esri. All rights reserved. For more information about Esri software, please visit www.esri.com.

We conducted a prospective observational cohort study to measure the effects of CATI response on cholera incidence in Northeast Nigeria with 2 response organizations. Solidarités International (SI) conducted CATIs in Borno, Yobe, and Adamawa states, and Action contre la Faim (ACF) operated in Borno and Yobe states (Fig 1). The Borno response was concentrated in the capital city of Maiduguri with a population of 800,000. The Adamawa response focused on the peri-urban towns of Jimeta and Yola with 600,000 people combined. The Yobe response was centered in the capital Damaturu, an agricultural area with 138,000 people.

### CATI description

SI and ACF followed similar standard operating procedures (SOPs) for their CATI responses. Cholera treatment centers (CTCs) provided CATI-response teams with line lists describing patients admitted to the CTC. According to the SOP, teams should provide every case household on the line list, and neighbor households within a 150m-radius, with a CATI response within 2 days of case arrival at the CTC. CATI teams should supply households with chlorine tablets, soap, and education materials. The SOP called for 2 months of soap and Aquatabs to case households and 1 month to neighbor households, adjusted for household size. Case households additionally received a 20 L jerrycan for water storage. Teams also conducted household-level activities including hygiene promotion, and disinfection of latrines and bedding by spraying these areas with a chlorine-solution for all case and neighbor households. CATIs did not include oral cholera vaccine (OCV) or antibiotic chemoprophylaxis (ACP), but teams did refer suspect cases to CTCs.

### Data collection and management

Data collection occurred from September to December 2021 and varied by state from: September 16 to December 16 in Borno (91 days), from September 18 to December 14 in Adamawa (87 days), and September 15 to November 15 in Yobe (61 days). During CATI response, enumerators embedded within CATI teams used Kobo Toolbox [29] (SI) and mWater [30] (ACF) to collect household-level data at each visited case and neighbor household. Data collected included demographics, observed existing WASH infrastructure, observed interventions provided, and global positioning system (GPS) coordinates. From the data, environmental and CATI variables were generated using R version 4.2.1 [31], RStudio version 2022.07.2+576 (Table 1) [32]. For WASH infrastructure, households with missing data were presumed to have the lowest level: unimproved water source, unimproved latrine, and no handwashing station. For CATI variables, households with missing data were presumed not to have received the intervention. All households with GPS precision >20 m, case households without visited neighbors, and neighbor households that were more than 300 m from a case were excluded from analysis.

**Table 1:** Environmental^a^ and CATI variables.

Variable	Category	Definition
Complete supplies–case	CATI	Case household received: • Aquatabs • Soap • Jerrycan
Complete supplies–neighbor	CATI	Neighbor household received: • Aquatabs • Soap
Complete activities	CATI	Household (case or neighbor) received: • Hygiene promotion • Bedding disinfection • Latrine disinfection
Ring coverage	CATI	Estimated proportion of households in ring that received a CATI. Numerator is the number of households in each 150-m radius ring that received a CATI. Denominator is the number of enumerated buildings in the ring.
Timeliness of response	CATI	Number of days between case arrival at CTC and CATI visit
CTC distance	Environmental	Distance in meters from case household to CTC, measured as the Euclidean distance using GPS coordinates.
Improved water source	Environmental	Existing household water source is borehole, rainwater, public tap/standpipe, protected well or spring, or is piped. All other water sources considered “unimproved.”
Improved latrine	Environmental	Existing household latrine is composting, flush, or pit with slab. All other latrine types considered “unimproved.”
Handwashing availability	Environmental	Household has an existing handwashing station with water and/or soap prior to intervention.

Variables for analysis were created from environmental and CATI data collected by research enumerators during each household visit. Variables were categorized as “CATI” or “environmental” depending on whether they were a measure of the CATI response or a potential environmental confounder.

^a^ Most humanitarian organizations like SI and ACF use humanitarian WASH standards, such as the Sphere Standards [35] or UNHCR for WASH Access [36], rather than Sustainable Development Goal standards [37]. Thus, the terms improved and unimproved are used herein. CATI, case-area targeted intervention; CTC, cholera treatment center; GPS, global positioning system. ACF, Action contre la Faim; SI, Solidarités International; WASH, water, sanitation, and hygiene.

To estimate the underlying population of the study area, households in CATI rings were manually enumerated and verified using ArcGIS Desktop (version 10.3.1) [27] and Google Earth [33]. The enumerated number of households was multiplied by the average study household size measured during data collection (5.5). Ordinary kriging [34] in ArcGIS was used to estimate continuous population counts for each state.

### Statistical analysis

Initial draft analysis plans included a more traditional approach of calculating incidence rates within each CATI ring and conducting unpaired *t* tests and Poisson regressions to evaluate the association between CATIs and cholera incidence. However, the complexity of real-life, uncontrolled CATI data in an urban humanitarian setting required adaptation to a more innovative spatiotemporal methodological approach.

Ultimately, prospective STSS were used to detect space-time clusters of cholera incidence during the study period and analyze CATIs’ effect on cholera clustering. Clustering is defined as disease incidence that exceeds baseline expectations if it were evenly distributed across population and covariates within a distinct scanning window. STSS detects clusters of cholera that have a geospatial relationship such that the area within a cluster represents excess risk as compared to the area outside of it. In the prospective approach, the statistic detects those clusters that are “active” or “emerging” on the most recent day of analysis.

Three models were run for each state: (1) an unadjusted model with case and population data; (2) an environmentally adjusted model adjusting for distance to cholera treatment centers, and existing WASH infrastructure including improved water source, improved latrine, and handwashing station; and (3) a fully adjusted model adjusting for environmental and CATI variables including supply of Aquatabs and soap, hygiene promotion, bedding and latrine disinfection activities, ring coverage, and response timeliness (Table 1). We conducted analyses for unadjusted models in RSaTScan [38] and analyses for environmental and fully adjusted models in SaTScan [39].

Data were aggregated to 150 m^2^ grid cells, approximating the 150 m CATI ring radius. We applied a discrete Poisson model with a maximum temporal window of 28 days (2 infectious periods) for space-time scans, starting analyses on day 14 of the outbreak, the maximum bacterial shedding and transmission risk period. Scans were then run on each subsequent day of the outbreak in each state: in Borno, 79 scans were conducted with an outbreak duration of 91 days; Adamawa saw 77 scans over 87 days; and in Yobe, 48 scans were run over 61 days. To ascertain statistical significance, we employed Monte Carlo simulations (*n* = 999) for each model, setting the threshold at *p* < 0.05.

Results characterize cholera cases in each location at each time period, accounting for background population. Covariates in the models control for specific effects. In the unadjusted model, statistically significant space-time clusters describe cholera cases that are not explained by population distribution alone. In the adjusted models, statistically significant space-time clusters describe cases not explained by population distribution and the covariates included in the model. Significant clustering represents excess risk that, when great enough, indicates an outbreak or epidemic. Cholera is endemic in Northeast Nigeria, and the declaration of an outbreak indicates excess risk beyond what would be expected based on historical data, environmental conditions, and population density in the affected areas. Nonsignificant clustering describes space-time associations that do not exceed incidence of cases beyond expectations when considering population density, random variation, and, for the adjusted models, covariates included in the models. Nonsignificant clustering signals incidence of cholera at non-outbreak levels.

We present descriptive statistics as medians with interquartile range (IQR) for continuous variables and percentages with counts for categorical variables. We describe the STSS results of the unadjusted and adjusted models as number of significant and nonsignificant clusters and mean relative risk (RR; defined as observed versus expected cholera incidence by population distribution), duration of clusters in days, and size of clusters with an RR >1.0 by number of 150 m^2^ grid cells. When the number of significant clusters permits, we provide cluster-specific details on time period, observed cases RR, size, and population. When the number of significant clusters prevents description in a table, we map findings over time. Visual representations demonstrate the temporal evolution of Borno clusters for RR, statistical significance (*p* < 0.05), duration, and recurrence interval (the number of days required to encounter the same expected number of clusters as observed clusters by chance). S1 Appendix provides details on STSS methodology, parameters, and additional visual representations showcase key findings over time in Adamawa and Yobe states.

### Ethical review

SI and ACF obtained Nigeria state-specific ethics approvals (SI: Borno SHREC Approval No. 050/2021; Yobe MOH/GEN/747/Vol. 1; Adamawa MENV/GEN/61/Vol.I/P.10, ACF: Borno SHREC Approval No. 052/2021; Yobe MOH/GEN/747/Vol. 1). Review from Johns Hopkins Bloomberg School of Public Health provided a determination of Public Health Surveillance (#14535) and non-research determination respectively. Adult participants provided informed verbal consent for participation.

## Results

### CATI case and neighbor households

**Fig 2:**
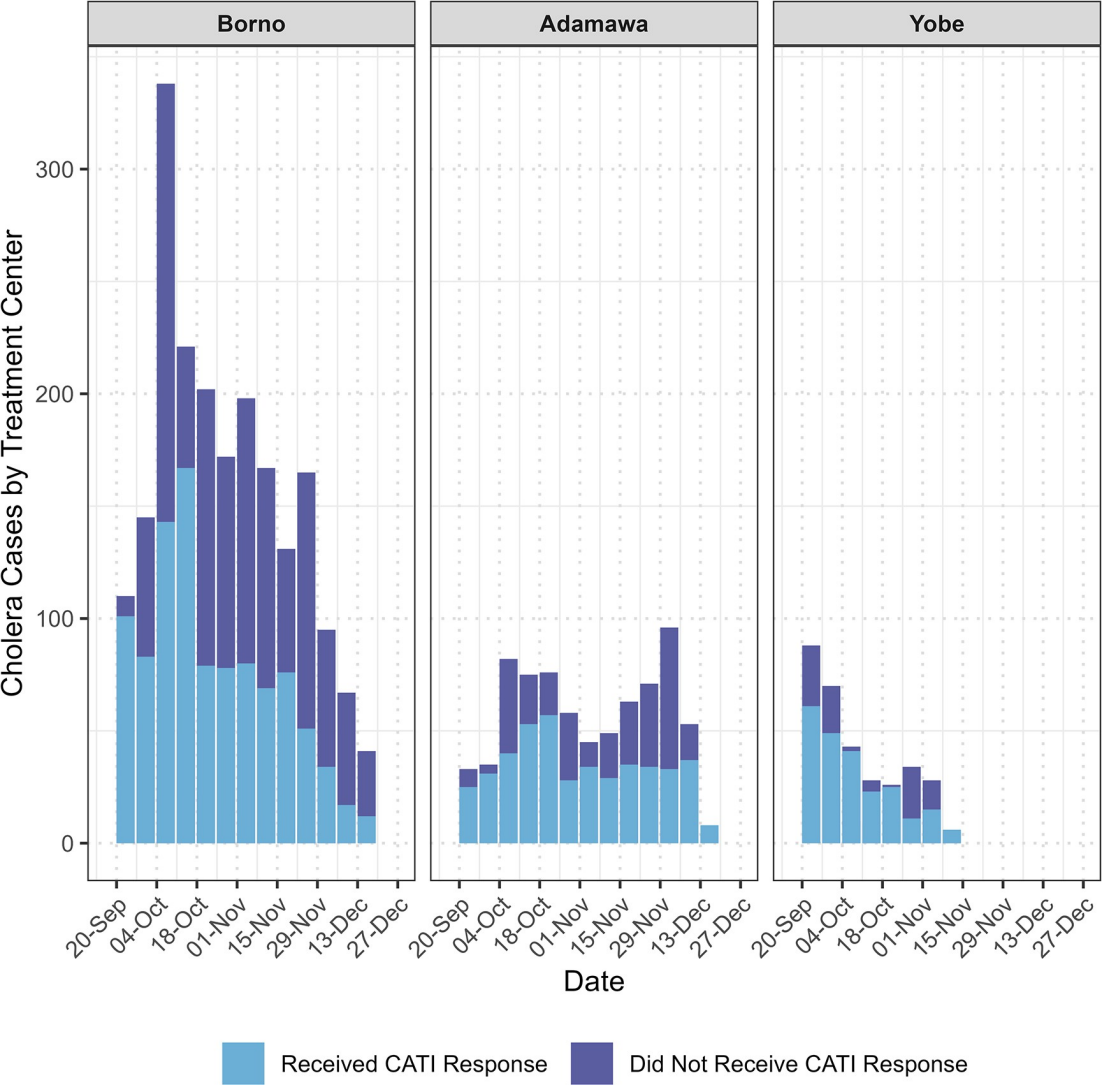
Epidemic curve of cholera case incidence and implementation of case area targeted interventions in Northeast Nigeria September 15–December 15, 2021. Confirmed and suspected cholera cases that arrived at a cholera treatment center were registered on line lists, which were then provided to CATI teams. The y-axis shows the total number of cholera cases registered at each state’s CTC. The area shaded in light blue indicates the number of line list cases that received a CATI. The area shaded in purple indicates the number of line list cases that did not receive a CATI. CATI, case-area targeted intervention; CTC, cholera treatment center.

Over the study period, 3,626 (suspect and confirmed) cholera cases reported to a CTC in the 3 states, of which 52.7% (*n* = 1,910) received a CATI response (Borno 46.6%, *n* = 1,139/2,442; Adamawa 60.0%, *n* = 451/752; Yobe 74.1%, *n* = 320/432; Fig 2). Of the 51,764 households visited by CATI teams, 1,287 (2.25%) declined intervention (3.42% in Borno, 0.09% in Yobe, and 2.81% in Adamawa), 3,060 (5.36%) had no one home (8.57% in Borno, 3.22% in Yobe, and 3.71% in Adamawa), and 1,001 (1.75%) were excluded for other reasons (0.07% in Borno, 0.13% in Yobe, and 3.43% in Adamawa). The analysis included 1,615 case and 42,982 neighbor households after data cleaning, with Borno accounting for 54.5% (*n* = 881) of case and 50.5% (*n* = 21,726) of neighbor households. Protected water sources were present in 95.7% (*n* = 21,646/22,607) of households in Borno, 69.4% (*n* = 7,865/11,330) in Adamawa, and 78.8% (*n* = 8,403/10,660) in Yobe. Improved latrines were present in 33.0% (*n* = 7,469/22,607) of households in Borno, 67.3% (*n* = 7,627/11,330) in Adamawa, and 89.6% (*n* = 9,554/10,660) in Yobe (Table 2). A basic handwashing station was present in 33.2% (*n* = 7,504/22,607) of households in Borno, 72.8% (*n* = 8,248/11,330) in Adamawa, and 49.6% (*n* = 5,290/10,660) in Yobe.

**Table 2:** Household and CATI characteristics by state, Northeast Nigeria, September 15 to December 25, 2021.

	Borno	Adamawa	Yobe	Total
**CATI Response** (*n*)^a^				
Total Registered Line List Cases	2,442	752	432	3,626
Line List Cases Received CATI (%)	1,139 (46.6)	451 (60.0)	320 (74.1)	1,910 (52.7)
Neighbors Received CATI	24,889	11,372	11,762	45,249
Total Case and Neighbors Received CATI	26,039	11,825	12,083	49,947
**Households Included in Analysis (*n*)**				
Cases	881	403	331	1,615
Neighbors	21,726	10,927	10,329	42,982
Total Case and Neighbors (%)^b^	22,607 (86.8)	11,330 (95.8)	10,660 (88.2)	44,597 (89.3)
**Household Characteristics** (% (*n*))				
Water Source Type				
* Protected*	95.7 (21,646)	69.4 (7,865)	78.8 (8,403)	85.0 (37,914)
* Purchased*	2.2 (505)	12.6 (1,423)	16.5 (1,758)	8.3 (3,686)
* Piped*	1.5 (335)	13.7 (1,557)	2.9 (304)	4.9 (2,196)
* Unimproved*	0.5 (121)	4.3 (485)	1.8 (195)	1.8 (801)
Improved Latrine	33.0 (7,469)	67.3 (7,627)	89.6 (9,554)	55.3 (24,650)
Handwashing Availability				
* Basic (Soap +/- Water)*	33.2 (7,504)	72.8 (8,248)	49.6 (5,290)	47.2 (21,042)
* Limited (Water)*	61.6 (13,918)	6.8 (770)	47.9 (5,109)	44.4 (19,797)
* None*	5.1 (1,157)	5.1 (577)	2.0 (218)	4.4 (1,952)
* Could Not Observe*	0.1 (28)	15.3 (1,735)	0.4 (43)	4.0 (1,806)
**CATI Characteristics** (Median (IQR))				
CATI Ring Coverage Percentage	4.0 (2.0, 11.0)	12.0 (7.0, 20.0)	20.0 (12.0, 39.0)	9.0 (4.0, 18.0)
CATI Ring Radius in m	50 (29, 102)	88 (60, 120)	78 (47, 137)	69 (35, 113)
Neighbors Visited per Case	12 (7, 35)	24 (13, 38)	28 (17, 44)	17 (9, 39)
Response Time in Days	2 (1, 4)	2 (1, 3)	0 (0, 1)	2 (1, 3)
Distance from Case to CTC in km	5.5 (3.3, 7.8)	3.2 (1.5, 5.6)	2.6 (1.6, 7.6)	4.3 (2.3, 7.5)
**CATI Implementation** (% (*n*))				
Aquatabs Distribution	55.8 (11,900)	31.4 (3,559)	81.0 (8,624)	55.6 (24,083)
Soap Distribution	47.2 (8,521)	18.9 (2,137)	37.9 (4,025)	36.7 (14,683)
Complete Supplies Distribution Case * (Soap + Aquatabs + Jerrycan)*	96.0 (816)	56.8 (229)	75.4 (248)	81.7 (1,293)
Complete Supplies Distribution Neighbor * (Soap + Aquatabs)*	44.3 (7,619)	17.5 (1,905)	33.2 (3,414)	33.7 (12,938)
Hygiene Promotion	99.9 (22,536)	65.8 (7,450)	99.3 (10,585)	91.1 (40,571)
Latrine Disinfection	99.9 (22,545)	100.0 (11,319)	99.4 (10,600)	99.8 (44,464)
Bedding Disinfection	99.9 (21,347)	100.0 (11,317)	84.5 (9,012)	96.2 (41,676)
Complete Activities *(Hygiene Promotion* *+ Bedding and Latrine Disinfection)*	94.1 (21,283)	65.8 (7,440)	84.2 (8,937)	87.1 (37,660)
Complete CATI Case * (Supplies + Activities)*	95.5 (805)	55.0 (221)	70.7 (232)	80.0 (1,258)
Complete CATI Neighbor * (Supplies + Activities)*	33.5 (7,571)	17.7 (1,889)	29.6 (3,359)	28.7 (12,819)

^a^ *n*, number; IQR, interquartile range; CATI, case-area targeted intervention.

^b^ Total Number of Case and Neighbor Households represents the total sample size from which the household characteristics, CATI characteristics, and CATI Implementation percentages are calculated.

Descriptive statistics on households and CATIs include the number of case and neighbor households, characteristics of those households, characteristics of the CATIs implemented, and supplies and activities provided by CATI teams.

### CATI response

Total median ring coverage was 9.0% (IQR 4.0%, 18.0%) and median ring radius was 69 m (IQR 35, 113). Both median ring coverage (4.0% [IQR 2.0%, 11.0%]) and median ring radius (50 m [IQR 29, 102]) were lowest in Borno. The median number of neighbor households visited per case was 17 (IQR 9, 39) with a median response time of 2 days (IQR 1,3). Borno teams visited fewer neighbor households per case (11 [IQR 7, 35]), had a longer median response time (2 days (IQR 1,4)), and traveled a greater distance from the CTC to the case (5.5 km [IQR 3.3, 7.8]) compared to Adamawa or Yobe.

Of the 44,597 households reached by CATIs in the 3 states, 33.7% (*n* = 12,938) received complete supplies, 87.1% (*n* = 37,660) received complete activities, and 33.5% (*n* = 12,819) received the complete CATI package (all supplies/activities defined in Table 1). Receipt of complete supplies for neighbors was more common in Borno (44.3% [*n* = 7,619/21,726]) than Adamawa (17.5%[*n* = 1,905/10,927]) and Yobe (33.2% [*n* = 3,414/10,329]). For activities, in Borno, 94.1% (*n* = 21,283/22,607) of neighbor households received the complete package. In Adamawa, all households received latrine and bedding disinfection, while 65.8% (*n* = 7,450/11,330) received hygiene promotion. In Yobe, 99.3% (*n* = 10,585/10,660) and 99.4% (*n* = 10,600/10,660) of households received both hygiene promotion and latrine disinfection, and 84.5% (*n* = 9,012/10,660) received bedding disinfection. The complete CATI package was provided to 33.5% (*n* = 7,571/22,607), 17.7% (*n* = 1,889/10,660), and 29.6% (*n* = 3,359/11,330) of households in Borno, Adamawa, and Yobe, respectively.

### Cholera clustering

#### Significant clustering

In the unadjusted model, we identified 291 significant clusters in Borno, 153 in Adamawa, and 128 in Yobe, indicating outbreak-level clustering beyond what is typically expected under normal conditions (Table 3). Adjusting for environmental factors like improved water source, improved latrine, handwashing station, and access to CTC, attenuated significant clustering considerably, with only 8 significant clusters in Borno, 0 in Adamawa, and 10 in Yobe. In Adamawa, when adjusting only for environmental factors, all the significant, outbreak-level clustering was accounted for. In the fully adjusted model, adjusting for both CATI and environmental factors eliminated all remaining significant, outbreak-level clustering in Borno and Yobe.

**Table 3:** Cholera Cluster Characteristics by State, Northeast Nigeria, Sept 15 to Dec 25, 2021.

	Unadjusted Model			Environmentally Adjusted Model ^c^			Fully Adjusted Model (Environmental + CATI) ^d^^,^^e^		
	Borno	Adamawa	Yobe	Borno	Adamawa	Yobe	Borno	Adamawa	Yobe
**Significant Clusters**								
**Total Clusters** ^**a**^	291	153	128	8	0	10	0	0	0
**Relative Risk** ^**b**^	8.0 (0.0, 87.6)	7.1 (0.0, 86.0)	7.1 (0.0, 40.2)	2.2 (1.8, 2.8)	-	5.5 (3.6, 8.3)	-	-	-
**Duration** ^**b**^ **(Days)**	22.7 (2.0, 26.0)	24.1 (2.0, 26.0)	23.6 (11.0, 26.0)	16.6 (5.0, 26.0)	-	19.1 (8.0, 26.0)	**-**	**-**	**-**
**Size** ^**b**^ **(150m**^**2**^ **Grid Cells RR>1)**	108.8 (1.0, 1,625.0)	196.0 (4.0, 569.0)	229.9 (5.0, 393.0)	24.6 (10.0, 98.0)	-	704.6 (474.0, 971.0)	**-**	**-**	**-**
**Observed Cases** ^**b**^	32.6 (0.0, 202.0)	27.1 (0.0, 75.0)	24.8 (0.0, 99.0)	56.8 (32.0, 92.0)	-	11.1 (7.0, 14.0)	**-**	**-**	**-**
**Expected Cases** ^**b**^	37.8 (0.1, 124.1)	23.6 (0.0, 48.1)	24.0 (0.2, 59.8)	29.9 (0.2, 59.8)	-	2.5 (0.9, 4.1)	**-**	**-**	**-**
**Non-Significant Clusters**								
**Total Clusters** ^**a**^	123	36	3	208	77	89	79	77	48
**Relative Risk** ^**b**^	30.8 (0.0, 404.3)	24.2 (0.0, 125.7)	116.8 (29.8, 252.5)	4.7 (0.2, 37.9)	4.5 (1.7, 11.1)	5.6 (0.3, 19.0)	3.1 (0.4, 10.5)	2.2 (1.4, 3.3)	4.7 (0.5, 12.0)
**Duration** ^**b**^ **(Days)**	21.1 (2.0, 26.0)	21.3 (5.0, 26.0)	21.3 (5.0, 26.0)	18.8 (2.0, 26.0)	18.9 (2.0, 26.0)	19.4 (2.0, 26.0)	15.7 (2.0, 26.0)	9.7 (2.0, 26.0)	18.9 (2.0, 26.0)
**Size** ^**b**^ **(150m**^**2**^ **Grid Cells RR>1 )**	11.6 (1.0, 76.0)	5.0 (4.0, 7.0)	7.3 (2.0, 15.0)	161.3 (1.0, 1,233.0)	492.8 (1.0, 3,085.0)	421.2 (4.0, 2,171.0)	24.7 (1.0, 527.0)	148.2 (1.0, 762.0)	178.9 (8.0, 629.0)
**Observed Cases** ^**b**^	4.3 (0.0, 13.0)	1.6 (0.0, 4.0)	2.7 (2.0, 4.0)	5.9 (2.0, 19.0)	5.9 (2.0, 19.0)	7.2 (2.0, 20.0)	11.1 (2.0, 65.0)	3.5 (2.0, 10.0)	3.9 (2.0, 15.0)
**Expected Cases** ^**b**^	8.4 (0.0, 35.9)	4.4 (0.0, 10.5)	0.1 (0.0, 0.1)	2.1 (0.2, 11.5)	2.1 (0.2, 11.5)	2.2 (0.1, 9.5)	9.5 (0.2, 50.0)	1.8 (0.6, 7.4)	1.5 (0.2, 8.7)

^a^ (n)

^b^ (Mean (Range))

^c^ The Environmentally adjusted model adjusted for existing WASH infrastructure: availability of improved water source, improved latrine, handwashing station, and distance to cholera treatment center (CTC).

^d^ The Fully adjusted (Environmental + CATI) model adjusted for environmental factors and CATI factors including complete supplies, complete activities, ring coverage, and response time.

^e^ CATI, case-area targeted intervention

Prospective space-time scan statistics yielded measures such as the number of cholera clusters and their relative risk, duration, size, and statistical significance, for each model in each state. The total clusters shows the number of significant and non-significant clusters by state over the epidemic. Relative risk measures the observed vs expected cholera incidence based on population distribution and assumption of independent distribution of events at a constant. The duration provides the average length of clusters in days. The size of the cluster in number of 150m^2^ grid cells with RR >1 indicates the mean number of 150m^2^ grid cells with relative risk >1 across all significant clusters.

In the unadjusted model, significant clusters carried high risk with a mean RR of 8.0 (range 0, 87.6), 7.1 (range 0, 86.0), and 7.1 (range 0, 40.2) in Borno, Adamawa, and Yobe, respectively. The environmentally adjusted model had lower mean RR in significant clusters with 2.2 (range 1.8, 2.8) in Borno and 5.5 (range 3.6, 8.3) in Yobe. As no significant clusters were detected in Adamawa, there was no excess risk to the population. Likewise in the fully adjusted model, as there were no significant clusters in any of the states, there was no excess risk.

In the unadjusted model, the mean duration of significant clusters was lower in Borno at 22.7 days (range 2.0, 26.0) than in Adamawa (24.1 [range 2.0, 26.0]) or Yobe (23.6 [range 11.0, 26.0]). When looking at the environmentally adjusted model, the mean duration was again lower in Borno at 16.6 days (range 5.0, 26.0) than Yobe at 19.1 days (range 8.0, 26.0). In Borno, the mean size of clusters, as measured by 150 m^2^ grid cells with an RR >1, was greater in the unadjusted model than the environmentally adjusted model (108.8 [range 1.0, 1,625.0] versus 24.6 [range 10.0, 98.0]). In Yobe, conversely, the mean size of clusters in the unadjusted model was smaller than in the environmentally adjusted model (229.9 [range 5.0, 393.0] compared to 704.6 [range 474.0, 971.0]). The mean number of observed to expected cases in the unadjusted model was 32.6: 37.8 in Borno, 27.1: 23.6 in Adamawa, and 24.8: 24.0 in Yobe, compared to 56.8: 29.9 in Borno and 11.1: 2.5 in Yobe in the environmentally adjusted model.

We summarize the results of the significant clusters from the environmentally adjusted model in Borno and Yobe in Table 4. In Borno state, cluster 1 started on September 15, while the other significant clusters began between October 6 and 7. Clusters 2 and 3 were the shortest at 5 days and cluster 8 the longest at 26 days. The RR ranged from 1.80 in cluster 8 to 2.75 in cluster 3. Cluster 1 covered the greatest surface area 87 grid cells which had RR >1. Cluster 3 covered the smallest surface area with 7 150 m^2^ cells with an RR >1. In Yobe, the earliest clusters began on October 1, followed by 2 clusters on October 2 and the remaining 6 on October 6. Cluster 1 was the shortest at 8 days and clusters 8 and 10 the longest at 26 days each. The RR ranged from 3.7 in cluster 10 to 8.26 in cluster 1. Clusters 6, 8, 9, and 10 covered the largest surface area at 9 150 m^2^ grid cells with an RR >1. Cluster 1 had the smallest surface area of 4 150 m^2^ grid cells with an RR >1.

**Table 4:** Environmentally adjusted model Emerging Space-Time Clusters in Borno and Yobe States, Northeast, Nigeria Sept 15 to Dec 25, 2021.

Cluster	Time Period	p-value	Observed	Expected	Observed / Expected	RR ^a^	150m^2^ Grid Cells RR>1	Cluster Population
**Borno**								
1	Sept 15–Oct 5	0.044	34	15.51	2.19	2.43	87	32,351
2	Oct 6–Oct 11	0.023	45	21.67	2.08	2.25	9	19,197
3	Oct 7–Oct 12	0.013	32	12.33	2.59	2.75	7	14,200
4	Oct 6–Oct 7	0.044	50	24.99	2.00	2.12	10	21,449
5	Oct 6–Oct 23	0.025	55	28.01	1.96	2.07	11	19,197
6	Oct 6–Oct 26	0.017	56	28.44	1.97	2.08	11	19,197
7	Oct 6–Oct 29	0.017	90	53.36	1.69	1.81	16	23,994
8	Oct 6–Nov 1	0.011	92	54.86	1.68	1.80	16	23,994
**Yobe**								
1	Oct 6–Oct 14	0.032	7	0.88	7.91	8.26	4	66,982
2	Oct 6–Oct 17	0.018	8	1.08	7.39	7.75	5	66,982
3	Oct 6–Oct 20	0.008	9	1.35	6.66	7.01	6	86,671
4	Oct 1–Oct 21	0.031	12	2.88	4.17	4.43	8	116,783
5	Oct 6–Oct 23	0.012	8	1.05	7.58	7.93	5	66,982
6	Oct 1–Oct 24	0.029	13	3.27	3.97	4.23	9	116,783
7	Oct 2–Oct 25	0.023	12	2.82	4.26	4.52	8	116,783
8	Oct 2–Oct 28	0.040	14	4.12	3.39	3.61	9	116,783
9	Oct 6–Oct 29	0.026	14	3.97	3.52	3.75	9	82,957
10	Oct 6–Nov 1	0.031	14	4.02	3.48	3.70	9	82,957

^a^ RR, relative risk

The Environmentally adjusted model adjusted for existing WASH infrastructure: availability of improved water source, improved latrine, handwashing station, and distance to cholera treatment center (CTC). The model resulted in eight significant clusters in Borno, ten in Yobe, and zero in Adamawa. The time period shows the start and end date of the significant clusters. The *p*-value indicates statistical significance of clusters as determined through Monte Carlo simulations. The observed/expected cases shows the measure of absolute risk. Relative risk measures the observed vs expected cholera incidence based on population distribution and assumption of independent distribution of events at a constant. The size in number of 150m^2^ grid cells with RR >1 indicates the total number of 150m^2^ grid cells with relative risk >1 present across all significant clusters. The cluster population indicates the total population residing in all significant clusters.

**Fig 3:**
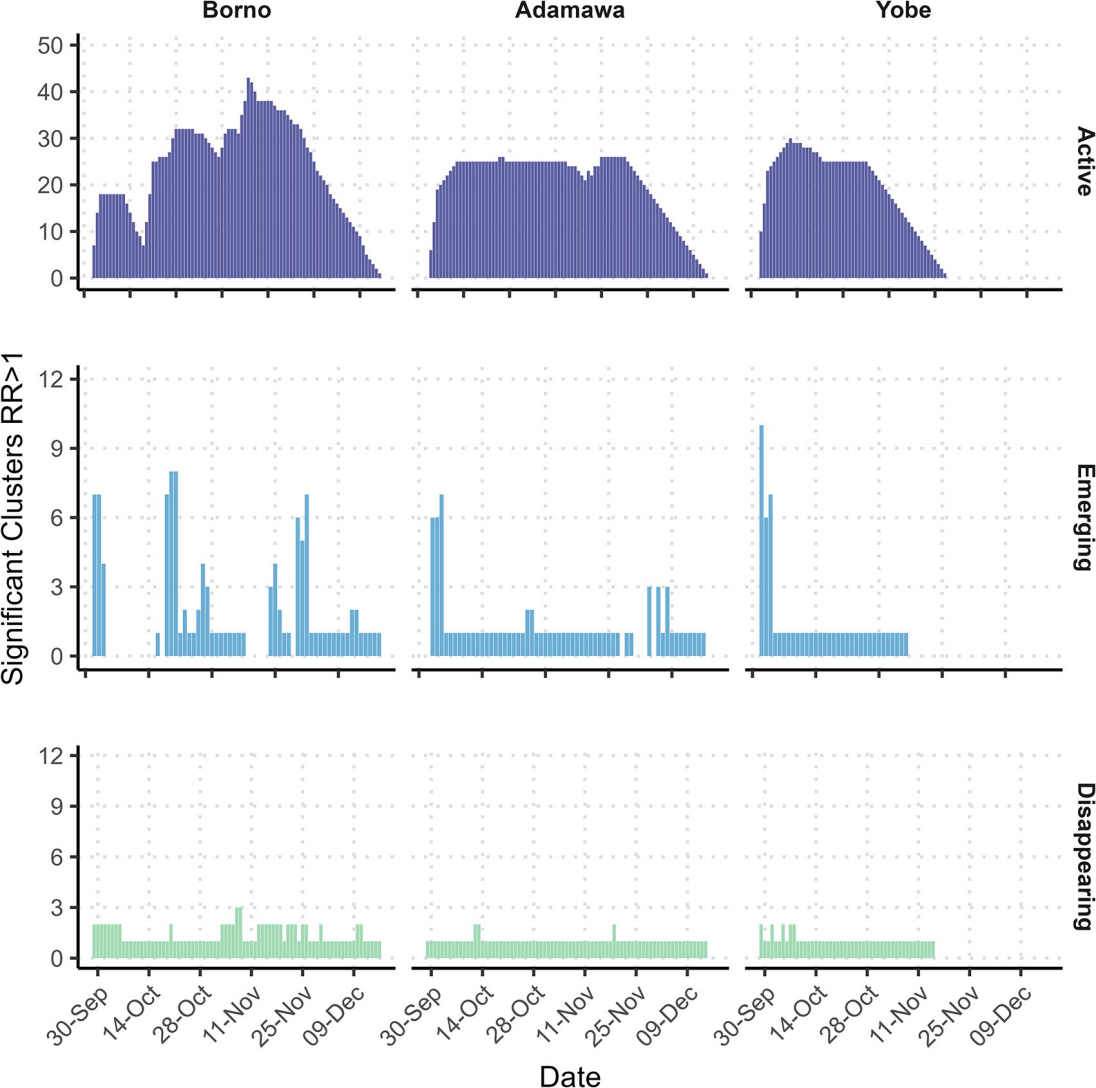
Unadjusted model active, emerging, and disappearing cholera clusters over outbreak period by state. The unadjusted model analyses considered the case and population data. The top panel in purple shows the number of statistically significant active cholera clusters by state over the epidemic. The middle panel in blue shows the number of statistically significant emerging cholera clusters and the bottom panel in green shows the number of statistically significant disappearing cholera clusters.

Due to the high number of significant clusters in the unadjusted model, we graphed key statistics over the outbreak period, showing the number of emerging and active clusters from the unadjusted model in the 3 states (Fig 3). Clusters with a start date on a particular day are “Emerging,” while clusters that began at an earlier date are “Active” and clusters with an end date are “Disappearing.” Other results from the unadjusted model such as mean RR, observed and expected cases, and total grid cells in clusters, over the outbreak period are included in S1 Appendix.

#### Nonsignificant clustering

Nonsignificant clustering was present across all models in all states, ranging from 3 in Yobe to 123 in Borno in the unadjusted model; from 77 in Adamawa to 208 in Borno in the environmentally adjusted model; from 48 in Yobe to 79 in Borno in the fully adjusted model (see Table 3). Nonsignificant clusters indicate cases of cholera within the expected range given endemicity and population density in the area and in adjusted models, after accounting for specific covariates. In the unadjusted model, most clustering was significant suggesting a cholera caseload at outbreak levels, with fewer clusters falling within what would be expected. In the environmentally adjusted model, more of the clustering was nonsignificant, meaning continued transmission and risk, though within the bounds of what would be expected given the population and covariates. In the fully adjusted model, in addition to elimination of significant clustering, nonsignificant clustering was reduced in Borno and Yobe states. In Adamawa, the number of nonsignificant clusters did not change between the environmental and the fully adjusted model, though the relative risk, duration, and size of clusters were all reduced. In Borno and Yobe states, relative risk, duration, and size of clusters were likewise reduced in the nonsignificant clusters.

#### Comparison of clustering between models

**Fig 4:**
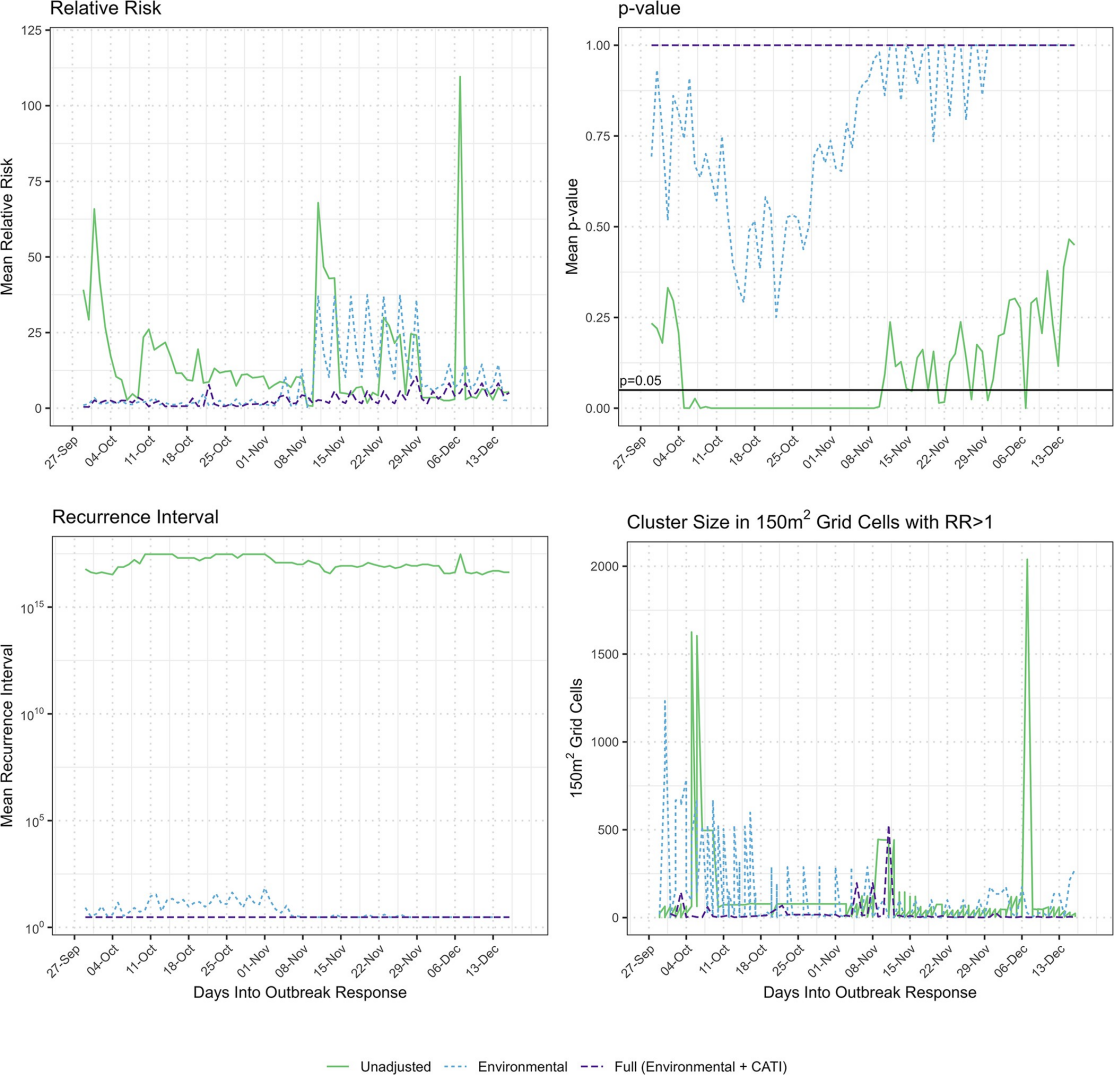
Comparison of unadjusted, environmental, and full CATI model on mean relative risk, mean significance, mean recurrence interval, and total size of significant and nonsignificant cholera clustering over time, Borno State, Nigeria. The Environmentally adjusted model (light blue, dotted line) adjusted for availability of improved water source, improved latrine, handwashing station, and distance to CTC. The Fully (Environmental + CATI) adjusted model (purple, dashed line) adjusted for the environmental factors and CATI factors including complete supplies, complete activities, ring coverage, and response time. The Unadjusted model (green, solid line) accounted for case count and population only. Relative risk measures the mean observed vs expected cholera incidence based on population distribution and assumption of independent distribution of events at a constant for each day of the epidemic. The *p*-value indicates the mean statistical significance of clusters as determined through Monte Carlo simulations for each day of the epidemic. Recurrence interval is a measure of how often an observed cluster would be the same size or larger by chance. It is presented as a mean for each day of the epidemic. The size in number of 150 m^2^ grid cells with RR >1 indicates the total number of 150 m^2^ grid cells with relative risk >1 present across all significant clusters for each day of the epidemic. CATI, case-area targeted intervention; CTC, cholera treatment center; RR, relative risk.

When comparing characteristics of significant and nonsignificant clusters, the fully adjusted model demonstrated consistently strong performance, indicating reduced risk of outbreak level case incidence. In Borno it had a lower RR, continuous insignificant *p*-value (≥0.05), smaller area of RR >1 as measured by 150 m^2^ grid cells, and lower recurrence interval over time, when compared to the unadjusted and environmentally adjusted models (Fig 4). In Borno, the environmentally adjusted model performed better than the unadjusted model though had higher levels of RR, particularly during the latter half of the outbreak, trends towards significant clustering, higher recurrence intervals in the first half of the outbreak, and larger areas of the cluster with RR >1 as measured by number of 150 m^2^ grid cells. Results for the full, environmental, and unadjusted models were similar for Adamawa and Yobe states, with graphs shown in S1 Appendix.

**Fig 5:**
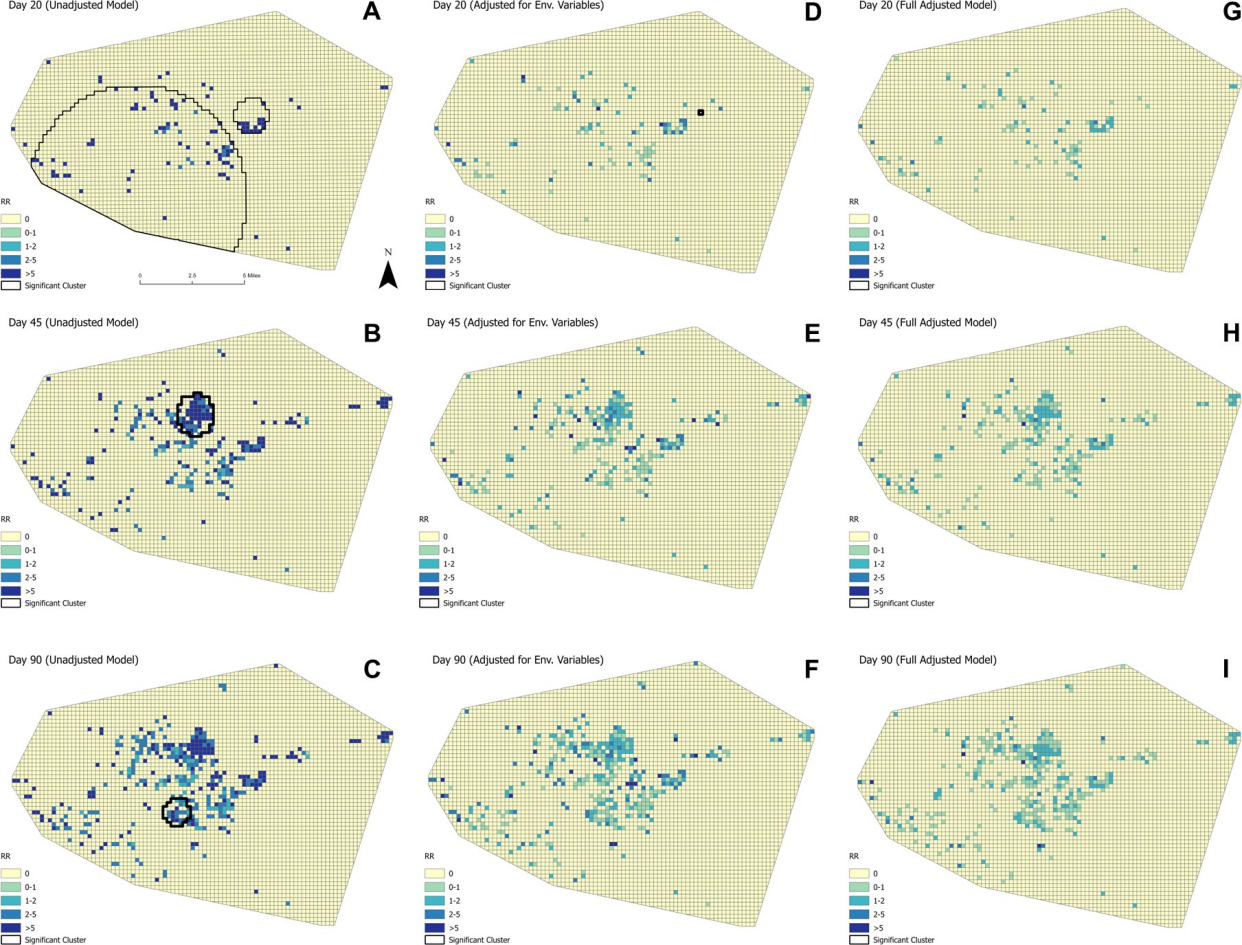
Mean RR of cholera incidence, Borno state, Nigeria. RR of clusters was calculated for each day in the study period. Mean relative risk of clusters at select days in the analysis (Day 20 [October 15, 2021], Day 45 [Octokber 30, 2021], Day 90 [December 14, 2021]) are presented. The unadjusted model (A–C) only accounts for case and population data. The environmentally adjusted (env.; D–F) model adjusts for environmental variables, including distance to CTCs and existing WASH infrastructure (improved water source, improved latrine, handwashing station). The fully adjusted model (Full Adjusted Model; G–I) adjusts for both environmental and CATI variables. CATI variables include complete supplies (household received Aquatabs, soap, IEC materials, and, if a case household, a jerry can), complete activities (household received hygiene promotion, latrine disinfection, and bedding disinfection), ring coverage (proportion of households in the ring that received a CATI), and response time (number of days between case arrival at CTC and CATI visit). Due to the cylindrical nature of STSS, clusters may include grid cells with lower-than-expected/no observed cases. To address this, the RR of each grid cell is represented. For ease of interpretation, cluster boundaries are not shown. Instead, RR of all grid cells across the study period are illustrated. CATI, case-area targeted intervention; CTC, cholera treatment center; IEC, information, education, and communication; RR, relative risk; STSS, space-time scan statistics; WASH, water, sanitation, and hygiene.

In Fig 5, choropleth maps illustrating mean RR over the study period from the unadjusted and fully adjusted models in Borno are displayed. An RR of 0 represents no observed cholera cases; RR >0 and RR <1 represents a lower-than-expected cholera risk, while RR >1 represents excess risk. In the unadjusted Borno model, high RR and significant clusters were concentrated in the north-central and central areas, and the periphery in various western and eastern areas. The fully adjusted model demonstrates a vast reduction in RR across grid cells with all clusters statistically nonsignificant, where only 3 grid cells exhibited an RR >7; most had an RR <1.

## Discussion

We evaluated the potential of CATIs to mitigate cholera transmission during the 2021 epidemic in conflict-affected Northeast Nigeria. Our findings suggest that CATIs played a crucial role in controlling the outbreak, showing CATIs attenuated cholera clustering and its associated risk in space and time better than environmental factors alone.

Across each state, we observed a substantial reduction in cholera clustering in the environmentally adjusted model, adjusting only for existing household WASH infrastructure and CTC access. In Adamawa, adjusting for the environmental factors eliminated significant clustering. This finding aligns with existing evidence that improved WASH infrastructure and access to care reduce the risk of cholera [40,41]. However, in conflict-affected areas, WASH infrastructure is difficult to build and maintain. Furthermore, in Borno and Yobe when adjusting only for the environmentally adjusted model, cholera still posed meaningful excess risk to the population. Significant clustering was present in 2 of 3 states indicating excess risk and caseload that continued to fuel the outbreak.

In the fully adjusted model, considering both existing environmental factors and CATI interventions, we observed an elimination of significant cholera clusters in Borno and Yobe, and thus a reduction in overall clustering, incidence, and excess risk in these states. The reduction in clustering extended to various associated measures, including cluster quantity, relative risk, statistical significance, likelihood of recurrence, size, and duration. The observed decrease in clustering in the fully adjusted model highlights the potential of these short-term interventions to disrupt wider transmission chains.

The fully adjusted model including CATI also reduced nonsignificant clusters in Borno and Yobe. In all states, including Adamawa, the inclusion of CATI reduced relative risk, duration, and size of clusters. While the fully adjusted model had few grid cells with an RR >1, none of which were statistically significant, these areas still carried risk and warranted continued monitoring to enable actors to control transmission before it reaches epidemic levels. Cholera is endemic in Northeast Nigeria, and as a bacterium that occurs in nature, it is not possible to reduce all risk of the disease. Nonsignificant clustering represents incidence that would be expected under usual, non-outbreak conditions. While outbreaks pose excess risk to the population, increasing illness and death, overburdening health services, and disrupting the economic and social stability of individuals and communities, endemic diseases also have negative effects on populations. Reducing disease incidence below expected levels will protect public health and benefit the quality of life and overall well-being of the community. The reduction of relative risk, duration, and size associated with CATI in nonsignificant clusters demonstrates its potential to reduce transmission outside of outbreak settings as well.

It is of note that the fully adjusted model including CATI attenuated risk across the states despite the exclusion of 2 highly effective health interventions, OCV and ACP. Currently, the global cholera vaccine stockpile is limited and OCV is distributed for mass campaigns only [42], making it challenging to obtain OCV for CATIs, despite its potential to reduce transmission [16,24]. While community administration of ACP is not recommended due to cost, contraindications, inability to prevent contamination, and potential to create antimicrobial resistance, evidence suggests that targeted administration reduces transmission [23,43] without increasing resistance [23,44]. More evidence on ACP in targeted approaches like CATIs is needed. If integrated into CATIs, expanded availability of prophylactic measures, particularly OCV, is likely to further decrease cholera risk [2]. Nevertheless, these findings underscore that CATIs with short-term WASH interventions can be highly effective in the absence of OCV and ACP.

Our research also provides information on ring size, timeliness, components, and population density of CATIs. A review of previous CATI implementation noted considerable variation in the types and spatiotemporal application of interventions [45]. While SOPs advised targeting households within a 150 m radius, the median CATI ring radius in all 3 states fell within 50 to 125 m. The finding suggests that reducing the recommended CATI ring radius from 150 m could optimize efficiency, potentially enabling teams to prioritize the more vulnerable households closer to the case, while still reducing risk and conserving resources. Further research is needed to refine optimal ring size in different contexts.

Early detection and rapid response are critical to prevent cholera clustering before cases grow and transmission becomes harder to contain [18,22]. CATI outbreak response was prompt in Borno and Yobe. In Borno, CATIs began 4 days after the outbreak was declared on August 31; in Yobe, CATIs were initiated as cases increased in mid-August. Adamawa also reported an increase in cases in mid-August but did not initiate CATIs until September 17. While early response is preferable, CATIs may still be effective later in an epidemic. In Haiti [46] and Yemen [14], implementation of CATIs began midway through the epidemic. In Cameroon [47] and South Sudan [14], CATIs showed the potential to contain remaining pockets of transmission at the end of an outbreak, when case load is reduced. This parallels our findings in the nonsignificant clusters, where caseload and clustering were reduced to expected levels, but implementation of CATI was associated with further reduction of risk.

The timeliness of each individual CATI is critical in a cholera outbreak due to the disease’s short incubation period [48] and potential hyper-infectious state [49]. Borno and Adamawa states had a median response time of 2 days between a case’s arrival at the CTC and CATI response, while Yobe teams usually responded the same day. Shorter response time in Yobe was likely influenced by lower caseloads, ease of locating rural households, and sparser population density, meaning less traffic and fewer buildings. Strong coordination and reliable logistics support are needed to ensure a timely response. In our research, rapid CATI implementation likely played a pivotal role in reducing cholera clustering.

The CATIs’ short-term, household-level WASH interventions also likely influenced CATIs’ associated effect on cholera clustering. Existing evidence has shown the effectiveness of many of these short-term measures outside of CATIs, including a controlled trial of soap distribution [25] and systematic reviews of point-of-use water treatment [50] and hygiene promotion [40]. Research has shown one-time disinfection is initially efficacious, but recontamination can occur quickly [51]. Overall, short-term WASH interventions play an important part in outbreak control, particularly in settings where long-term maintenance of WASH infrastructure is challenging. More research is needed to identify the most effective CATI interventions in different contexts.

Previously published qualitative research from this study and reviews by UNICEF and Médecins Sans Frontières identified implementation challenges that may mediate CATI effectiveness such as high caseloads, incomplete line lists, stockouts, inadequate staffing, insufficient transportation, household refusals, security constraints, and high population density [14,52,53]. In Borno, the high population density resulted in cholera incidence that was 3 and 5 times greater than in Adamawa and Yobe, respectively, and translated into more households in a 150 m radius and increased travel time. Stockouts were common across states as reflected in the low percentage of households that received complete supplies.

Many of these challenges can be addressed through strong epidemic preparedness, including strategic staffing, advanced planning, pre-positioning of supplies, and strong logistics [18,20]. Proactively training WASH and health staff on contextualized SOPs, including how to address anticipated challenges, will ensure they are prepared before an outbreak occurs. Coordination among community members, healthcare providers, NGOs, and government agencies will leverage actors’ respective strengths and resources to address challenges and optimize CATI effectiveness. Local health promotion teams can raise awareness, address concerns, and coordinate with actors and the community to mitigate difficulties. Other community interventions, such as enhanced case detection via community-based surveillance would expand CATIs reach beyond line lists, improve detection for mild and moderate cases, and provide opportunities for early and rapid control. Our study also indicates the potential to use space-time scan statistics, like the methods in this study, as a form of real-time surveillance to identify areas of high transmission.

Our study has a number of limitations. Selection bias was evident as cholera case incidence was only recorded for cases that reached the CTC and received a CATI. The study design precluded ascertainment of GPS coordinates of cases that did not receive CATIs. A large proportion of cholera cases exhibit mild or no symptoms and only the more severe cases typically seek care at a CTC [54]. Thus, the study focused on CATI association with cholera cases that sought treatment at a CTC and received a CATI. This analysis focuses on the effects of complete CATIs and does not analyze or otherwise assign significance to individual interventions. In addition, data collection tools only recorded whether a certain supply was provided and did not record quantity. It is possible that teams provided fewer supplies than planned if facing supply shortages, which would underestimate the findings. Remote data collection driven by the COVID-19 pandemic and security concerns limited supervision, potentially leading to measurement errors. Data limitations prevented the authors from analyzing free chlorine residual, a crucial household water quality measure affecting cholera transmission. As a prospective cohort study, we could not establish causality between CATI response and cholera clustering, leaving room for unmeasured confounding and alternative explanations of our findings. Some factors, such as household socioeconomic status, were not measured. Others, such as climate, rainfall, and health-seeking behavior had minimal variability between states, which prevented comparative analyses due to their geographic proximity and the study design which only included cholera cases that sought care at a CTC.

The study also has several notable strengths. We assessed CATI effectiveness in real-world conditions, reflecting outcomes from actual implementation rather than idealized, unsustainable scenarios derived from controlled studies [25]. The analysis provided a comprehensive examination of cholera incidence across the study area, offering a direct and objective measure of CATI attenuation of severe disease incidence. Daily data collection enabled precise and detailed detection of spatial and temporal variations in disease patterns over the three-month period. The large sample size enhanced robustness, minimizing the likelihood that chance findings or data fluctuations influenced results. The variation in geographic context, encompassing urban, peri-urban, and rural settings, suggests findings may have broad applicability. Innovative application of time scans and data aggregation to 150 m^2^ grid cells enabled precise cluster detection over space and time. With this approach, we identified clusters with significant and nonsignificant associations and isolated CATI effects by comparison with alternative models.

Future research is needed to confirm our findings in different contexts and with alternative interventions, including the use of OCV or ACP, and activities to improve longer-term WASH infrastructure, such as installation of improved latrines. Additionally, it should guide operational strategies by determining the most effect CATI supplies and activities, the optimal CATI ring radius and coverage in different contexts, the role for community-based activities, the effect on intermediate outcomes like household water quality and cost-effectiveness [19]. These steps are vital for efficient resource allocation and improvement in CATI strategies.

Cholera remains a global public health concern affecting vulnerable populations. This research suggests CATIs have the potential to reduce cholera transmission in conflict settings. As cholera outbreaks generally occur in settings without water and sanitation infrastructure, our results should not be interpreted as prioritizing CATIs over infrastructure. Ideally, every household worldwide should have safely managed water and sanitation access that inhibits cholera transmission. Until such time, CATIs provide a valuable role in preventing transmission in cholera outbreaks. Overall, our findings have meaningful public health significance. Given the current global surge in cholera cases, particularly in conflict and fragile settings where WASH access is often limited, our results offer strong justification for implementing and scaling up CATIs in such contexts.

## Supporting information

S1 STROBE Checklist STROBE checklist. (DOCX)

S1 Appendix Supplementary material. Additional figures and details on the space-time scan statistics (STSS) are presented in this appendix.
(DOCX)

## Abbreviations

ACF: Action contre la Faim
ACP: antibiotic chemoprophylaxis
CATI: case-area targeted intervention
CFR: case fatality rate
CTC: cholera treatment center
GPS: global positioning system
IQR: interquartile range
OCV: oral cholera vaccine
RR: relative risk
SI: Solidarités International
SOP: standard operating procedure
STSS: space-time scan statistics
WASH: water, sanitation, and hygiene
