# Supplementary material for: Case-area targeted interventions during a large-scale cholera epidemic: A prospective cohort study in Northeast Nigeria
Source: PLoS Med. 2024 May 10;21(5):e1004404. doi: 10.1371/journal.pmed.1004404 (PMC11149837; doi:10.1371/journal.pmed.1004404)
Supplement: S1 Appendix — Additional figures and details on the space-time scan statistics (STSS) are presented in this appendix. (DOCX) [file pmed.1004404.s002.docx]

**Supplementary Material:**

**A prospective study of case-area targeted interventions during a large-scale cholera epidemic in Northeast Nigeria in 2021**

**Table of Contents**

Space-Time Scan Statistics (STSS) Details ………………………………. 1

Additional Figures ……………………………………………….………………… 3

**Space-Time Scan Statistics (STSS) Details**

STSS have been widely used to monitor outbreaks of infectious disease. The prospective version of STSS can detect “active” or emerging clusters of disease during the most recent time period of analysis (e.g., days, weeks, etc.), disregarding clusters that may have existed previously as shown in the retrospective STSS variant. In essence, the prospective STSS utilizes moving cylinders that scan our study areas for potential space-time clusters of cholera. The base of the cylinder is the spatial scan while the height reflects the temporal scan, where the base of each cylinder is centered on the geographic centroids of each defined location (I.e., grid cells). Each cylinder is expanded until a user-defined maximum spatial and temporal upper bound is reached, where each cylinder is a potential cluster. This process is repeated around each location (I.e., grid cells) until each has a complete set of potential space-time clusters. In other words, an unknown number of scanning cylinders are generated around each grid cell centroid until the maximum spatial and temporal thresholds are met, while the observed and expected cholera cases are computed within each cylinder, derived from the total cases and underlying population within each grid cell. A grid cell and time period (I.e., day) is part of a potential cluster if its geographic centroid is located within a particular cylinder. A portion of a grid cell may intersect with a cylinder, but it’s centroid point must be within for it to be considered for that iteration.

For this study, we set the maximum spatial scan to 10% of the population at-risk to account for groups of grid cells that may contain excess risk of cholera, while avoiding the detection of clusters that are too large to interpret the results in an effective manner. The temporal scan was set to 28 days, or two incubation periods of cholera after consulting with subject matter experts. We selected a discrete Poisson model where the number of cholera cases are assumed to follow a Poisson distribution according to the population of each study area in this analysis. The null hypothesis states that the model reflects a constant risk with an intensity µ, proportional to the at-risk population. The alternative hypothesis states that the number of observed cholera cases exceed the number of expected cases derived from the null model, which is defined as the elevated risk within a cylindrical scanning window. The expected number of cholera cases µ is computed as the population within a grid cell multiplied by the total rate of cholera in the study area (I.e., p*C/P). Our model assumes that the population is static within each location across the study period.

Next, a maximum likelihood ratio test is used to detect scanning windows with an elevated risk for cholera, defined as:

Where L(Z) is the likelihood function for cylinder Z, and L0 is the likelihood function for the null hypotheses; nz is the total number of cholera cases in a cylinder; µ(Z) is the total number of expected cholera cases in cylinder Z; N is the total number of observed cases for the entire study area; and u(T) is the total number of expected cases in the study area across all time periods. The function identifies an elevated risk within a cylinder when the likelihood ratio (LLR) is >1, that is(equation). The cylinder with the highest LLR is the most likely cluster, while secondary clusters are still reported in order of their LLR. Monte Carlo simulations (n=999) are then run to assess the statistical significance of each space-time cluster. Each simulation is given the same number of cases as the true, observed dataset and the likelihood is then computed. Therefore, n=999 LLRs are computed for each candidate space-time cluster which represents the distribution of the LLR under the null hypothesis of constant risk across space and time. Clusters are significant if p < 0·05. Note that the models are run each subsequent day until the final day of the study period is reached to properly examine the evolution and space-time dynamics of cholera clusters.

Since the RR of cholera is not homogenous within each cluster, because they may contain numerous grid cells, we also report and visualize the RR for each grid cell that belongs to a cluster. The RR for each grid cell that belongs to a cluster is defined as:

$${RR}_{i}=\frac{(\frac{c_{i}}{e_{i}})}{[\frac{\left( C-c_{i} \right)}{\left( C-e_{i} \right)}]}$$

Where $c_{i}$ is the total number of cholera cases in a grid cell *i*; $e_{i}$ is the total number of expected cases in a grid cell *i*; and C is the total number of cases in the study area *A*. The clusters also contain an overall RR, but this is divided by the risk outside of the cluster. The recurrence interval is an output statistic that is unique to the prospective STSS approach, representing the likelihood of the cluster occurring again by chance and forgoing the need to correct for multiple testing such as the Bonferroni adjustment. A recurrence interval value is essentially the inverse of the p-values. For example, a recurrence interval value of 30 (days) suggests that it may take another 30 days for that particular cluster to appear again. The higher the recurrence interval, the more likely the cluster was anomalous and corresponding to an outbreak of high excess risk.

We compute both unadjusted and adjusted STSS models for each study area and time period. The unadjusted models simply consider both cases and population. For the covariate adjustment models, the expected number of cholera cases are computed the same as the unadjusted model, but now includes a covariate category i, where:

$$E\left[ c \right]=\sum_{k} E[c_{k}]=\sum_{k} p_{k}*(\frac{C_{k}}{P_{k}})$$

Parameters $c_{k}$, $p_{k}$, $C_{k}$, and $P_{k}$ are defined the same way as the non-covariate model, but for covariate category *k.*As such, the adjusted STSS models search for clusters that cannot be explained by those covariates alone. For this study, if the size and magnitude (e.g., relative risk, LLR, recurrence interval, etc.) decreases in the adjusted models, then there is evidence that the CATI interventions successfully reduced cholera transmission in particular areas (grid cells) and time periods (days).

**Additional Figures**


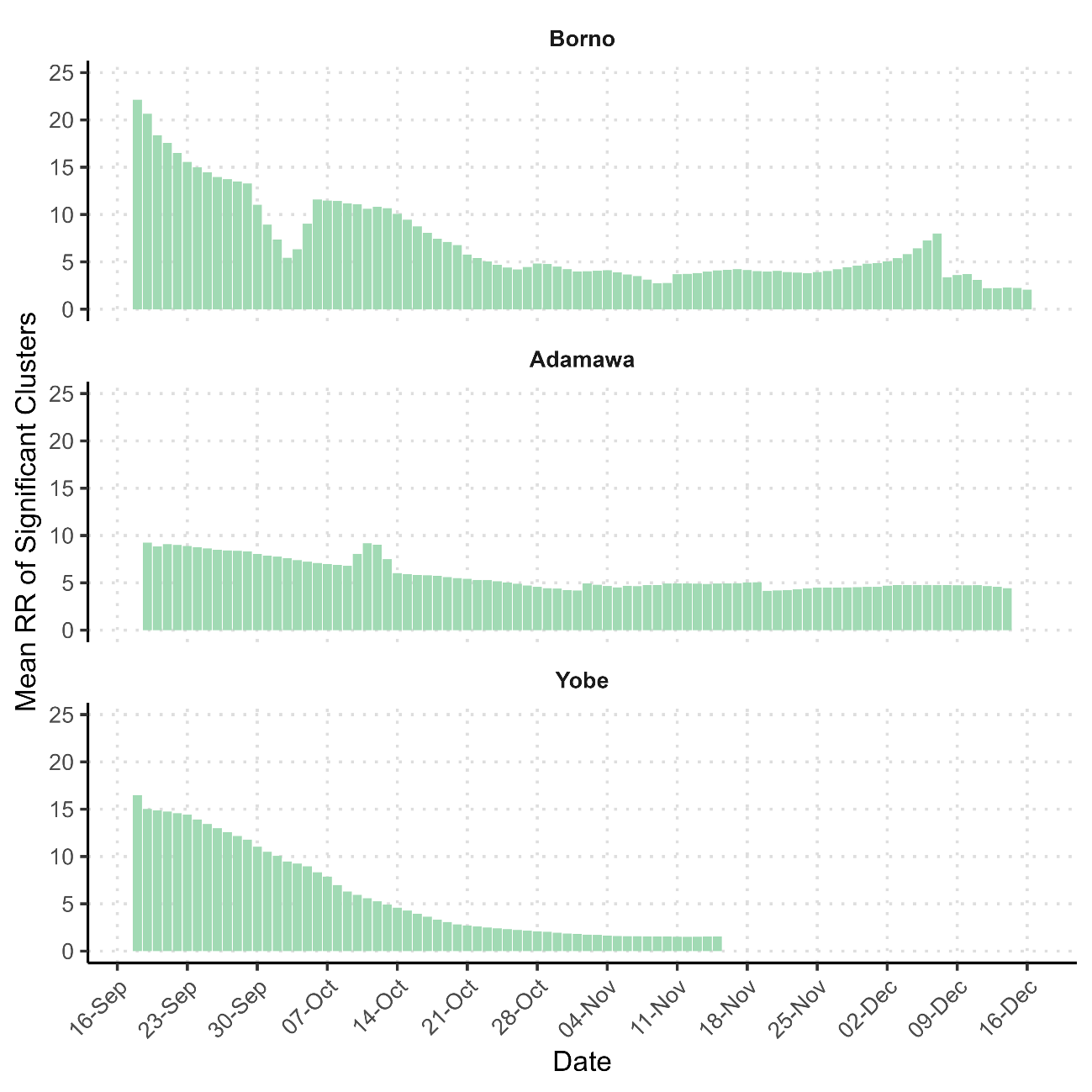


**Supplemental Fig 1. Unadjusted model, mean relative risk of significant clusters over time, by state.** *Relative risk measures the mean observed vs expected cholera incidence based on population distribution and assumption of independent distribution of events at a constant for each day of the epidemic, when only considering population and case data (unadjusted model).*


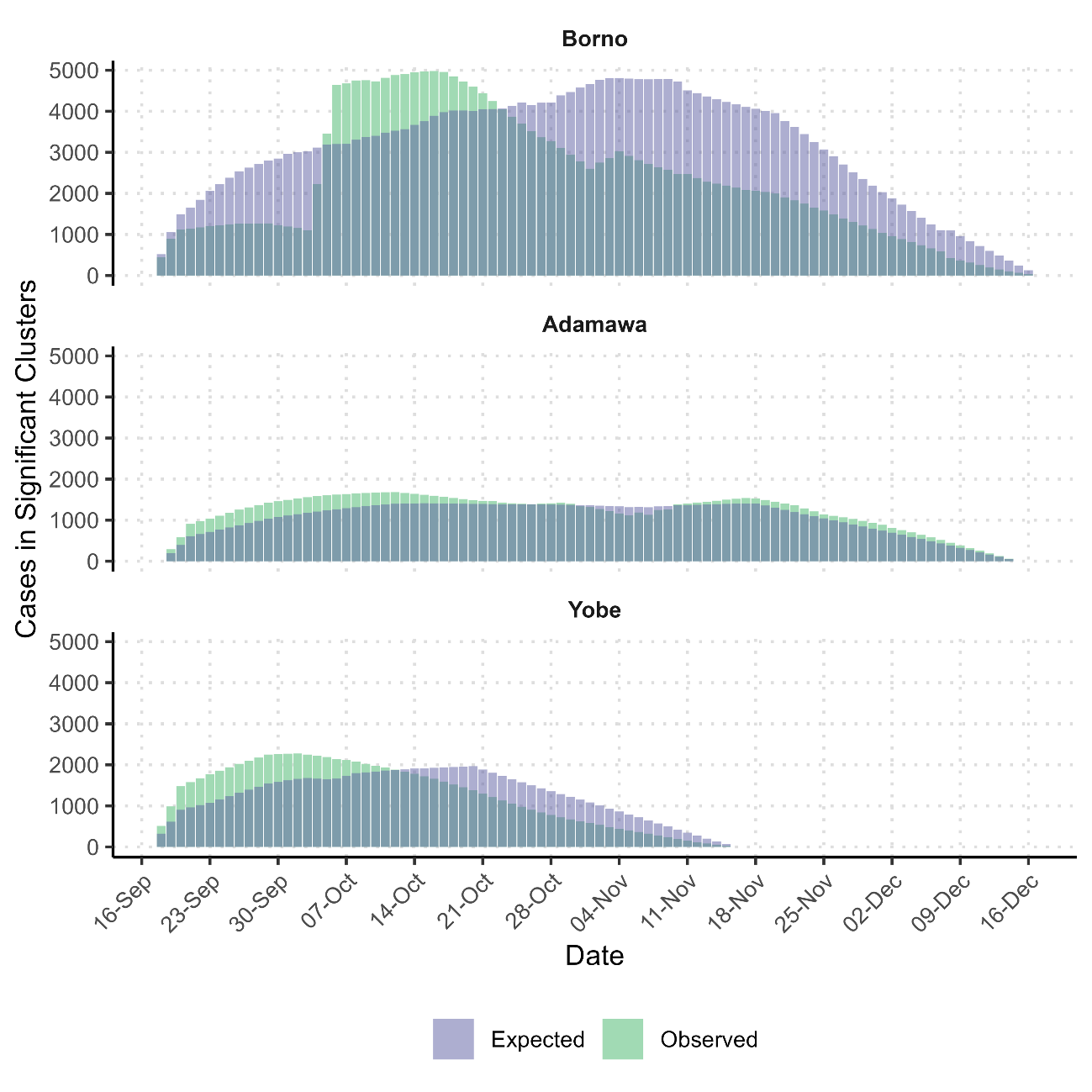


**Supplemental Fig 2. Unadjusted model, number of cases in significant clusters over time, by state.**

This figure shows the number of expected (purple; based on population) vs. observed (green) cases in statistically significant clusters for each day of the epidemic, when only considering population and case data (unadjusted model).


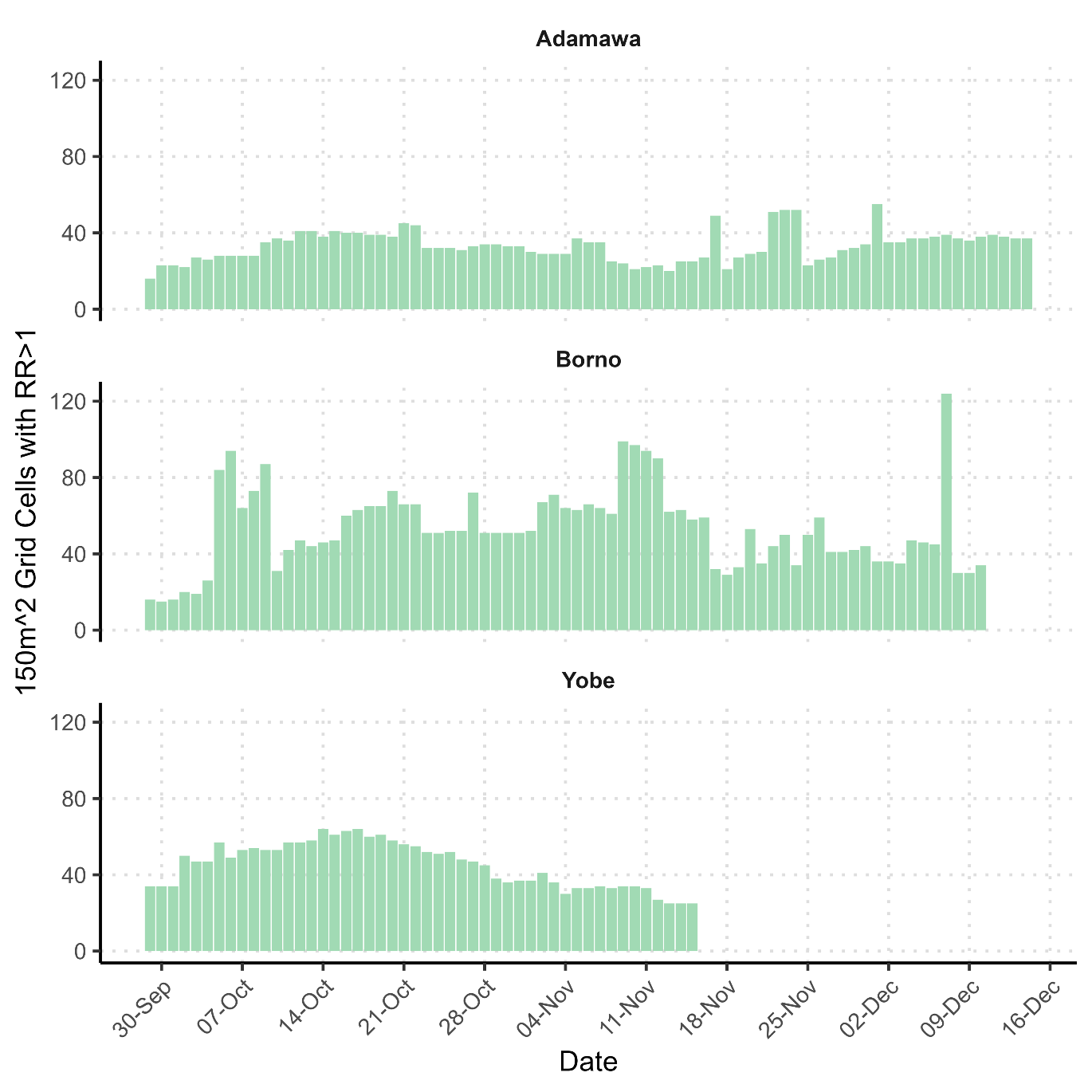


**Supplemental Fig 3. Unadjusted model, number of grid cells with RR>1.**

*The size in number of 150m^2^ grid cells with RR >1 indicates the total number of 150m^2^ grid cells with relative risk >1 present across all significant clusters for each day of the epidemic*, *when only considering population and case data (unadjusted model).*


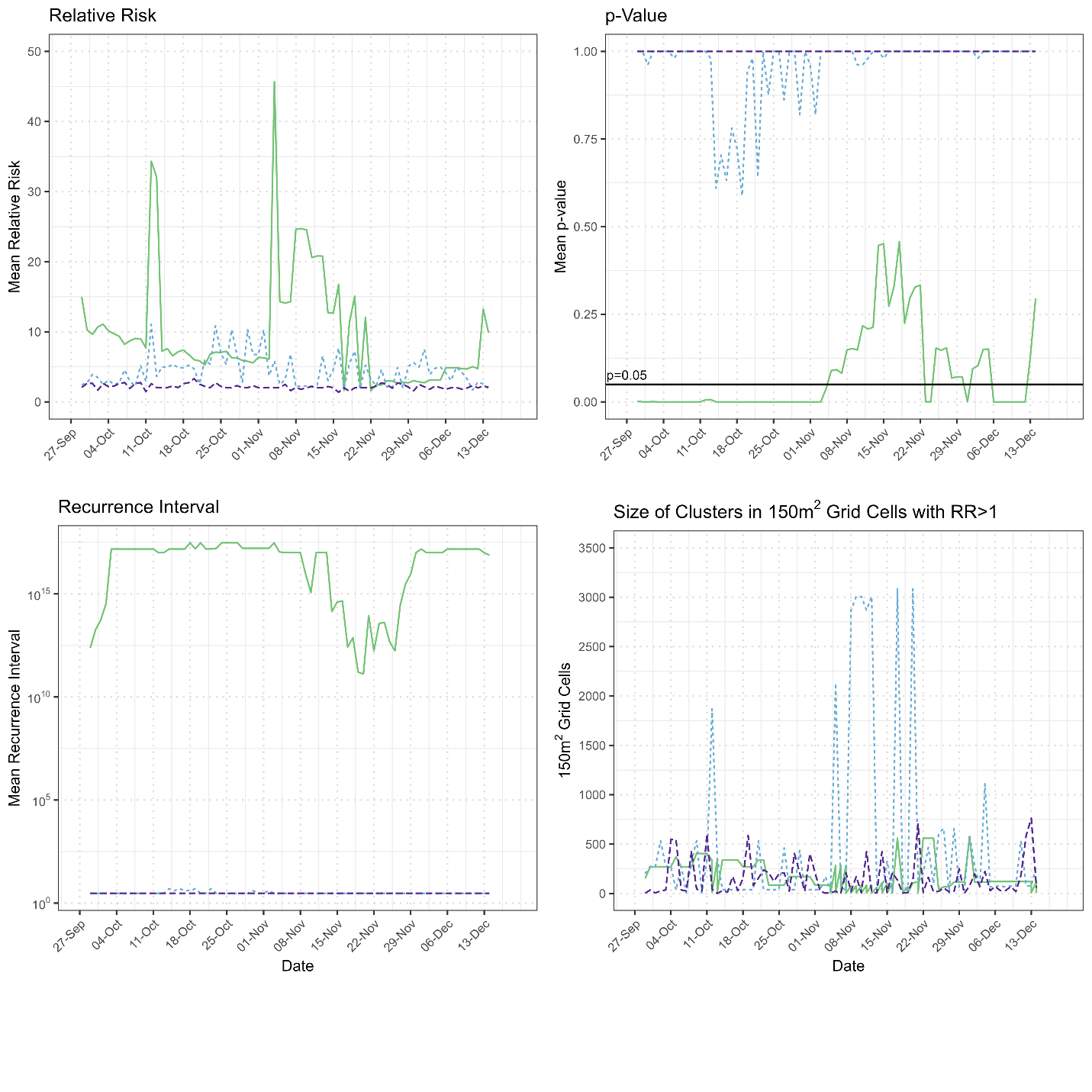


**Supplemental Fig 4. Impact of CATI on cholera clustering over time, Adamawa State, Nigeria.**

*The Environmentally adjusted model (light blue, dotted line) adjusted for availability of improved water source, improved latrine, handwashing station, and distance to CTC. The Fully (Environmental + CATI) adjusted model (purple, dashed line) adjusted for the environmental factors and CATI factors including complete supplies, complete activities, ring coverage, and response time. The Unadjusted model (green, solid line) accounted for case count and population only. Relative risk measures the mean observed vs expected cholera incidence based on population distribution and assumption of independent distribution of events at a constant for each day of the epidemic. The p-value indicates the mean statistical significance of clusters as determined through Monte Carlo simulations for each day of the epidemic. Recurrence interval is a measure of how often an observed cluster would be the same size or larger by chance. It is presented as a mean for each day of the epidemic. The size in number of 150m^2^ grid cells with RR >1 indicates the total number of 150m^2^ grid cells with relative risk >1 present across all significant clusters for each day of the epidemic.*


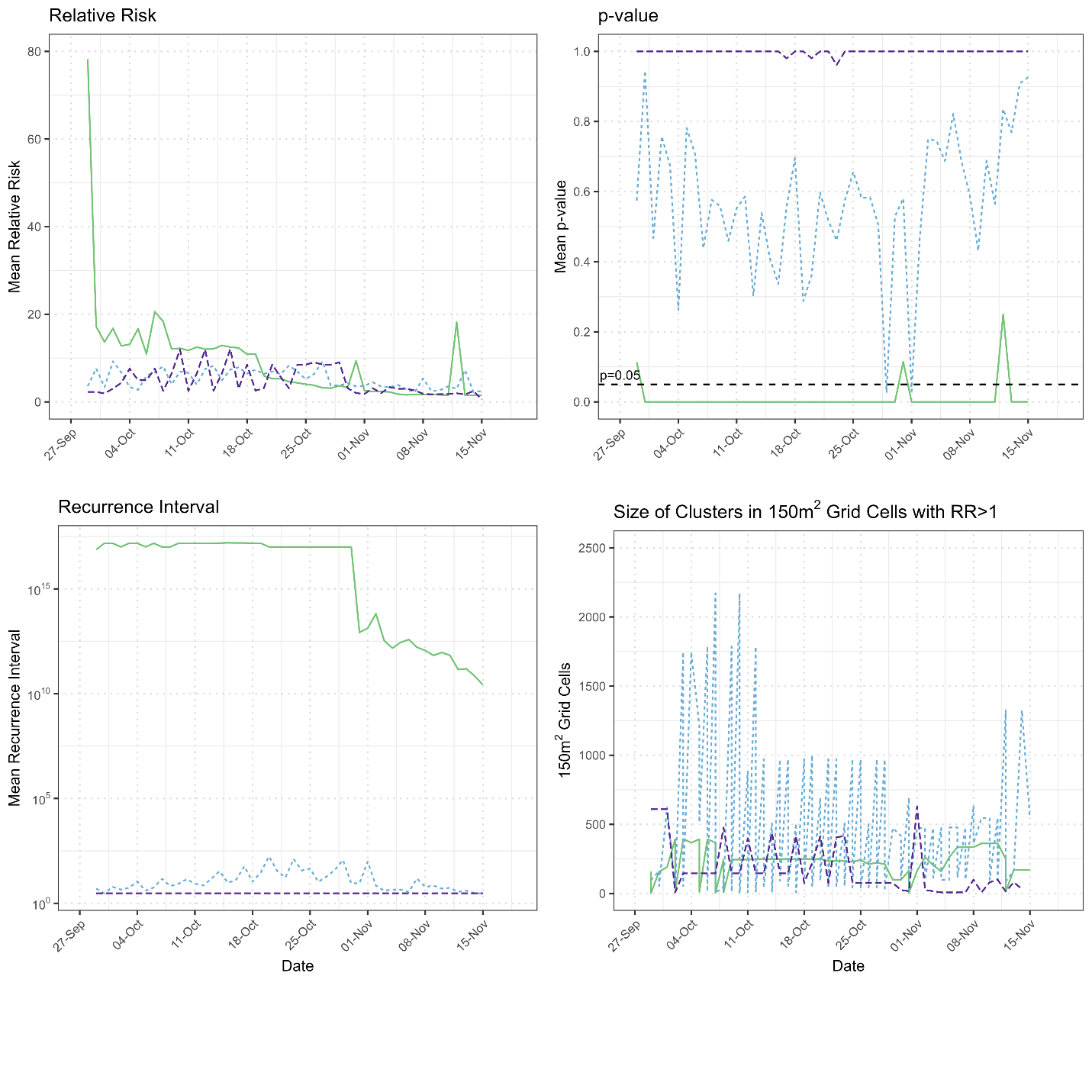


**Supplemental Fig 5. Impact of CATI on cholera clustering over time, Yobe State, Nigeria.**

*The Environmentally adjusted model (light blue, dotted line) adjusted for availability of improved water source, improved latrine, handwashing station, and distance to CTC. The Fully (Environmental + CATI) adjusted model (purple, dashed line) adjusted for the environmental factors and CATI factors including complete supplies, complete activities, ring coverage, and response time. The Unadjusted model (green, solid line) accounted for case count and population only. Relative risk measures the mean observed vs expected cholera incidence based on population distribution and assumption of independent distribution of events at a constant for each day of the epidemic. The p-value indicates the mean statistical significance of clusters as determined through Monte Carlo simulations for each day of the epidemic. Recurrence interval is a measure of how often an observed cluster would be the same size or larger by chance. It is presented as a mean for each day of the epidemic. The size in number of 150m^2^ grid cells with RR >1 indicates the total number of 150m^2^ grid cells with relative risk >1 present across all significant clusters for each day of the epidemic.*


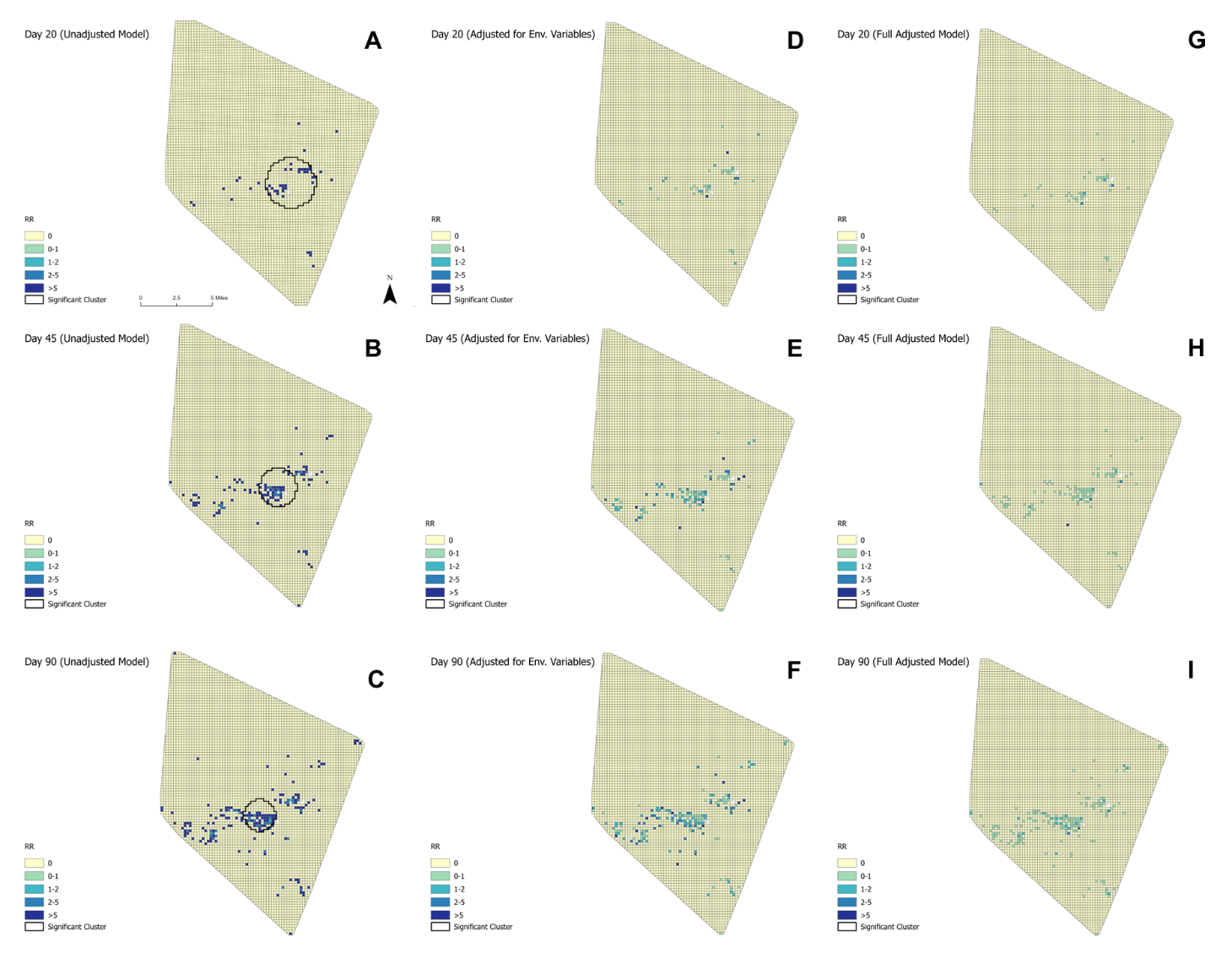


**C**

**Supplemental Figure 6. Mean Relative Risk (RR) of Cholera Incidence, Adamawa State, Nigeria.**

Relative risk (RR) of clusters was calculated for each day in the study period. Mean relative risk of clusters at select days in the analysis (Day 20, Day 45, Day 90) are presented. The unadjusted model (A-C) only accounts for case and population data. The environmentally adjusted (env.; D-F) model adjusts for environmental variables, which include distance to cholera treatment centers (CTC) and existing water, sanitation, and hygiene (WASH) infrastructure (improved water source, improved latrine, handwashing station). The fully adjusted model (Full Adjusted Model; G-I) adjusts for both environmental and case-area targeted intervention (CATI) variables. CATI variables include complete supplies (household received Aquatabs; soap; information, education, and communication [IEC] materials; and, if a case household, a jerry can), complete activities (household received hygiene promotion, latrine disinfection, and bedding disinfection), ring coverage (proportion of households in the ring that received a CATI), and response time (number of days between case arrival at CTC and CATI visit). Note that due to the cylindrical nature of space-time scan statistics (STSS), clusters may include grid cells with lower-than-expected/no observed cases. To address this, the relative risk (RR) of each grid cell is represented. For ease of interpretation, cluster boundaries are not shown, only RR of all grid cells across the study period.


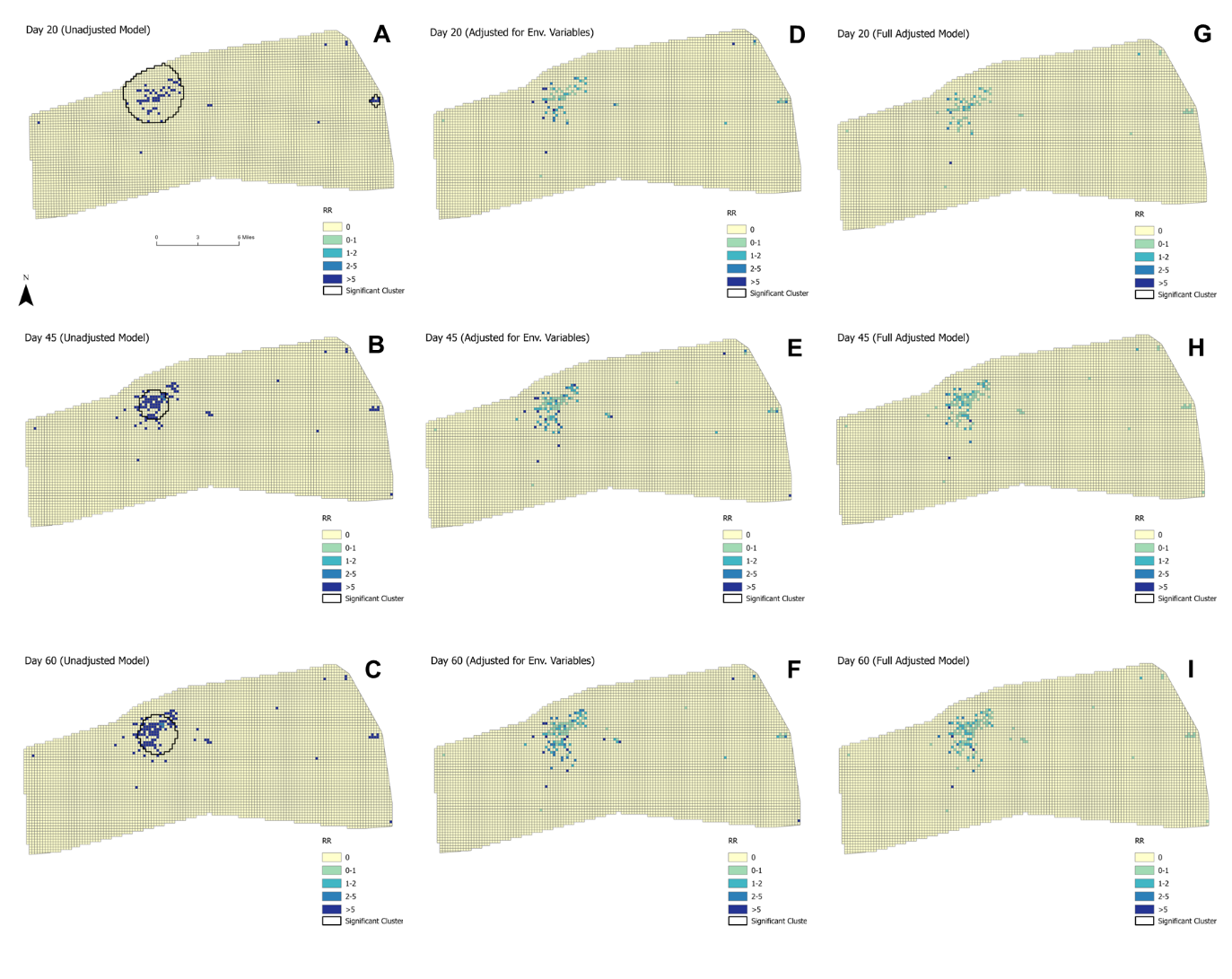


**Supplemental Figure 7. Mean Relative Risk (RR) of Cholera Incidence, Yobe State, Nigeria.**

Relative risk (RR) of clusters was calculated for each day in the study period. Mean relative risk of clusters at select days in the analysis (Day 20, Day 45, Day 90) are presented. The unadjusted model (A-C) only accounts for case and population data. The environmentally adjusted (env.; D-F) model adjusts for environmental variables, which include distance to cholera treatment centers (CTC) and existing water, sanitation, and hygiene (WASH) infrastructure (improved water source, improved latrine, handwashing station). The fully adjusted model (Full Adjusted Model; G-I) adjusts for both environmental and case-area targeted intervention (CATI) variables. CATI variables include complete supplies (household received Aquatabs; soap; information, education, and communication [IEC] materials; and, if a case household, a jerry can), complete activities (household received hygiene promotion, latrine disinfection, and bedding disinfection), ring coverage (proportion of households in the ring that received a CATI), and response time (number of days between case arrival at CTC and CATI visit). Note that due to the cylindrical nature of space-time scan statistics (STSS), clusters may include grid cells with lower-than-expected/no observed cases. To address this, the relative risk (RR) of each grid cell is represented. For ease of interpretation, cluster boundaries are not shown, only RR of all grid cells across the study period.
